# Membrane Fusion‐Mediated Cytosolic Delivery of Threose Nucleic Acids via Homotypic Nanoparticles Overcomes Drug Resistance in Triple‐Negative Breast Cancer

**DOI:** 10.1002/advs.76100

**Published:** 2026-06-12

**Authors:** Wei Zheng, Tristan Juin Han Chang, Xinchao Li, Zhongqi Zhou, Chung Tin, Kenward Vong, Pierre Karam, Pik Kwan Lo

**Affiliations:** ^1^ Department of Chemistry and State Key Laboratory of Marine Environmental Health Kowloon Tong Hong Kong China; ^2^ Department of Biomedical Engineering City University of Hong Kong Kowloon Tong Hong Kong China; ^3^ Department of Chemistry The Hong Kong University of Science and Technology Kowloon Hong Kong China; ^4^ Department of Chemistry American University of Beirut Beirut Lebanon; ^5^ City University of Hong Kong Chengdu Research Institute Chengdu China; ^6^ Key Laboratory of Biochip Technology, Biotech and Health Care Shenzhen Research Institute of City University of Hong Kong Shenzhen China

**Keywords:** biomimetic, drug resistance, homotypic, membrane fusion, TNA

## Abstract

Triple‐negative breast cancer (TNBC) remains lethal due to its aggressive molecular heterogeneity and drug resistance. We report a biomimetic nanoplatform (PLL/TNA_AKT2_@CM NPs) integrating biostable threose nucleic acid (TNA) with a donor‐derived cell membranes (CMs) “cloak” for subtype‐specific therapy. By complexing TNA_AKT2_ antisense oligonucleotides with poly‐L‐lysine (PLL) and coating them with TNBS‐subtype membranes (MDA‐MB‐468 or MDA‐MB‐231), we achieve potent homotypic affinity. PLL/TNA_AKT2_@468CM NPs exhibited significant enhanced uptake in donor‐matched basal‐like 1 cells compared to heterotypic TNBC, non‐TNBC and normal epithelial cells. Mechanistically, these nanoparticles internalize via a rapid membrane‐fusion, bypassing endosomal entrapment for direct cytosolic delivery. This facilitates robust silencing of the AKT2 oncogene, achieving a **∼**70% protein knockdown and outperforming conventional transfection reagents. In a drug‐resistant MDA‐MB‐468 xenografts, systemic administration led to superior tumor accumulation, effective AKT2 knockdown, and significant tumor regression via the p21/Caspase‐3 apoptotic axis, without systemic toxicity. This versatile “plug‐and‐play” strategy addresses tumor heterogeneity and endosomal sequestration, providing a transformative paradigm for targeted nucleic acid delivery in refractory cancers.

## Graphical abstract

This study introduces a biomimetic “nanofusion” platform that integrates the biostability of threose nucleic acids (TNA) with homotypic cell‐membrane cloaking to combat drug‐resistant TNBC. By leveraging a non‐canonical membrane‐fusion pathway for direct cytosolic delivery, the platform bypasses endosomal sequestration. To achieve potent AKT2 silencing and significant tumor regression, offering a versatile framework paradigm for precision genetic medicine.

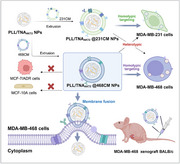

## 1 Introduction

Triple‐negative breast cancer (TNBC) is an aggressive and heterogeneous subtype of breast cancer that accounts for 15%–20% of all diagnosed cases. It is defined by its negative status for expression of three key receptors: estrogen receptor (ER), progesterone receptor (PR), and human epidermal growth factor receptor 2 (HER2) [1]. Compared with other breast cancer subtypes, TNBC is characterized by a high degree of malignancy and invasiveness, a high rate of early recurrence and visceral metastasis, and limited treatment options, collectively making it one of the most clinically aggressive breast cancer subtypes [2].

The standard treatment for TNBC typically involves surgical excision, often followed by chemotherapy and/or radiotherapy. Despite these treatments, many TNBC patients still face a high risk of relapse and mortality. This is primarily due to the limitations of traditional chemotherapy, which include limited efficacy, severe systemic side effects, poor treatment response, and acquired drug resistance [3, 4]. In recent years, significant progress has been made with the approval of targeted therapies. This includes the use of poly (adenosine diphosphate) ribose polymerase (PARP) inhibitors, such as Olaparib [5], for patients with specific BRCA mutations. Additionally, immunotherapy options, including PD‐L1 inhibitors such as Pembrolizumab [6], are available for patients whose tumors express high levels of PD‐L1. Crucially, these advanced therapeutic options are only accessible to patients who meet the clinical criteria. Since these patients represent only a minority of all TNBC cases, there remains a pressing and unmet need within the medical community to explore and develop novel treatment modalities that can effectively address the challenges posed by TNBC for the broader patient population [7].

Gene therapy has recently emerged as a promising new therapeutic strategy for treating TNBC [8]. Over the past decade, RNA interference (RNAi) therapy, which encompasses the use of small interfering RNA (siRNA), microRNA (miRNA), and long noncoding RNAs (IncRNAs), has been recognized as a powerful therapeutic approach. RNAi can target TNBC‐associated genes, including CDK1 [9] and WEE1 [10] for cell cycle regulation, AKT, EGFR, BRD4 [11], and surviving [12] for proliferation, SFRP1 [13], PDCD4 and HoxD10 [14] for tumor suppression, and LINK‐A [15], TGF𝛽1 [16], Notch 1 [17], PKM2 [18], for metabolism, thereby interfering with signaling pathways associated with TNBC development and progression. Furthermore, combining traditional chemotherapy drugs with RNAi therapy has demonstrated synergistic effects in suppressing TNBC tumors, offering a multifaceted treatment strategy that can enhance efficacy and address the disease's complexity [19, 20]. However, RNA molecules are susceptible to degradation by enzymes in the bloodstream and are unable to pass through the hydrophobic cell membrane efficiently on their own. To overcome these delivery barriers, various nanocarriers, including cell‐penetrating peptide (CPP) vesicles [21], liposomes [22, 23], cationic polymer nanoparticles [24], metallic nanoparticles [25], silica nanoparticles [26], and single‐wall nanotubes [27], are utilized to encapsulate and transport RNA payloads for TNBC therapy. These nanocarriers have demonstrated excellent protective capability in preclinical settings. However, they often lack intrinsic targeting properties. Therefore, additional functional ligands, such as aptamers [28, 29], peptides [30], small molecules, and antibodies [31], must be chemically conjugated to the surfaces of these nanocarriers. The addition of these ligands significantly increases the design complexity, preparation time, and overall cost of the therapeutic system [32]. Even though RNA molecules are successfully protected during circulation and cellular internalization, they still face intracellular obstacles that hinder their function, primarily lysosomal trapping and poor enzymatic resistance before reaching their molecular targets.

To overcome the inherent limitations of natural nucleic acids in clinically settings, chemically modified synthetic oligonucleotides, such as phosphorothioate (PS) oligonucleotides [33, 34] and locked nucleic acids (LNA) [35] have been extensively investigated as antisense oligonucleotides (ASO). These modifications aim to stabilize the therapeutic cargo while silencing TNBC‐related mRNAs or long non‐coding RNAs (*Inc*RNAs). With this landscape, we have recently demonstrated that threose nucleic acid (TNA) serves as a uniquely versatile biomaterial for both diagnostic and therapeutic applications [36]. Unlike natural genetic material, synthetic TNAs possess an exceptional biocompatibility profile; they do not elicit pathological immune responses or induce structural damage, particularly within the renal system [37]. From a biochemical prospective, TNAs exhibited superior binding affinity toward complementary RNA compared to DNA, making them ideal candidates for high‐precision antisense applications [38, 39, 40]. Furthermore, TNAs are characterized by remarkable enzymatic resistance, sustained stability across a wide pH range, and resilience in human or fetal bovine serum, even under prolonged storage at room temperature [41]. Crucially, our previous work confirmed that sequence‐designed TNAs can successfully target therapeutic genes, such as the AKT gene, to suppress tumor cell proliferation in vivo for the treatment of TNBC [42]. This platform offers a cost‐effective and scalable alternative for cancer therapy due to its straightforward production and inexpensive reagents. However, despite their chemical robustness and safety [43, 44], the therapeutic translation of TNA remains bottlenecked by poor, cell‐line‐dependent internalization. This limitation is particularly pronounced in refractory, drug‐resistant TNBC subtypes, such as MDA‐MB‐468 and HCC‐1806 [42, 45, 46]. Furthermore, conventional delivery vehicles, such as cationic lipids or polymers, often exacerbate these issues by inducing significant systemic cytotoxicity and off‐target effects [45, 46].

**SCHEME 1:**
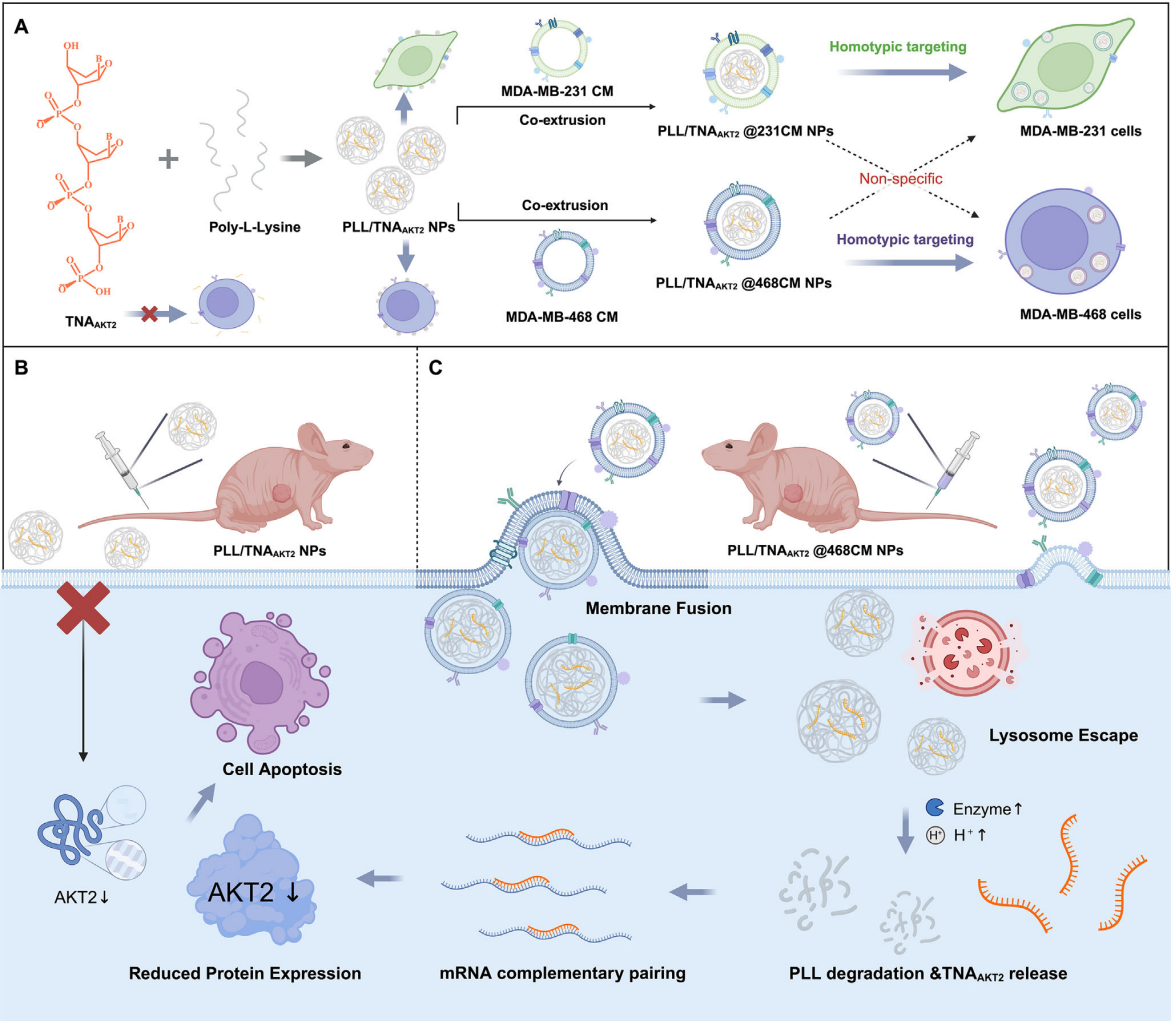
Biomimetic PLL/TNA_AKT2_@CM nanoparticles for TNBC‐targeted gene therapy. (A) Fabrication process: PLL/TNA_AKT2_ core formation via electrostatic self‐assembly, followed by coating with TNBC cell‐derived membranes (MDA‐MB‐468 or MDA‐MB‐231) via mechanical extrusion. (B) In vivo mechanism: Following intravenous injection, homotypic targeting enables selective tumor accumulation of membrane‐coated NPs. Membrane fusion‐mediated internalization bypasses endosomal trapping, allowing direct cytoplasmic delivery of TNA_AKT2_ for gene knockdown.

To address the delivery hurdles associated with TNAs and enhance subtype‐specific targeting, we demonstrate for the first time that a biomimetic “cloak” can resolve these delivery challenges. “Top‐down” cell membrane (CM) coating has emerged as a robust biointerfacing strategy for delivering diverse nucleic acid payloads including plasmid DNA, messenger ribonucleic acid (mRNA), small interfering RNA (siRNA), microRNA, and immunostimulatory CpG oligonucleotides. This approach effectively mitigates the biological barriers presented by the inherent negative charge, hydrophilicity, and large molecular size of nucleic acids [47, 48, 49]. To date, most CM‐coated platforms derived from red blood cells, cancer cells, and macrophages for TNBC treatment rely on classical endocytic pathways [50, 51, 52, 53]. However, this reliance typically subjects the therapeutic payload to the degradative endo‐lysosomal environment, significantly limiting cytosolic bioavailability. While membrane fusion has been suggested as a superior alternative, existing studies often lack comprehensive mechanistic proof and stringent homotypic specificity [54]. While the CM coating dictates the initial bio‐interaction with target cells, the encapsulated cargo's stability is determined by the assembly of the nanoparticle core. To facilitate the effective delivery of genetic materials across cell membranes, cationic polymers including polyethyleneimine (PEI) [55] and poly (lactic‐co‐glycolic acid) (PLGA) [56] are widely used. In this study, we selected PLL [57] to form complexes with therapeutic TNAs due to its superior biocompatibility, low toxicity, and ease of modification [58, 59]. This resulted in the development of a new targeted nanomedicine, named PLL/TNA_AKT2_@CM nanoparticles, engineered for TNBC subtype differentiation and in vivo gene therapy. We fabricated these nanoparticles by loading TNA‐based antisense drugs (TNA_AKT2_) into PLL cores, followed by clocking with phospholipid membranes derived from specific TNBC cell lines (MDA‐MB‐468 or MDA‐MB‐231) to exploit homotypic recognition (Scheme 1). Our results demonstrated that the cell membrane coating enables a staggering 100‐fold increase in TNA uptake in donor‐matched cancer cells compared to native TNA_AKT2_. These biomimetic PLL/TNA_AKT2_@MDA‐MB‐468 nanoparticles (PLL/TNA_AKT2_@468CM NPs) exhibited preferential targeting in vitro toward the basal‐like 1 subtype (MDA‐MB‐468) over mesenchymal stem‐like cells (MDA‐MB‐231), non‐TNBC lines and normal mammary epithelial cells. Crucially, the PLL/TNA_AKT2_@CM platform bypasses the traditional endosomal pathway via a membrane‐fusion mechanism, directly delivering the antisense agents into the cytoplasm. This direct access facilitated a profound inhibitory effect on the target AKT2 oncogene at both the mRNA and protein levels, far outperforming conventional transfection methods. In vivo, the CM‐coated NPs demonstrated robust accumulation within MDA‐MB‐468 tumor tissues without the need for auxiliary transfecting agents. This targeted delivery led to significant tumor regression and a marked decrease in AKT2 expression, successfully promoting apoptotic responses and suppressing tumor proliferation.

## 2 Results and Discussion

### 2.1 Fabrication and Characterization of PLL/TNA_AKT2_ Core Nanoparticles

**FIGURE 1:**
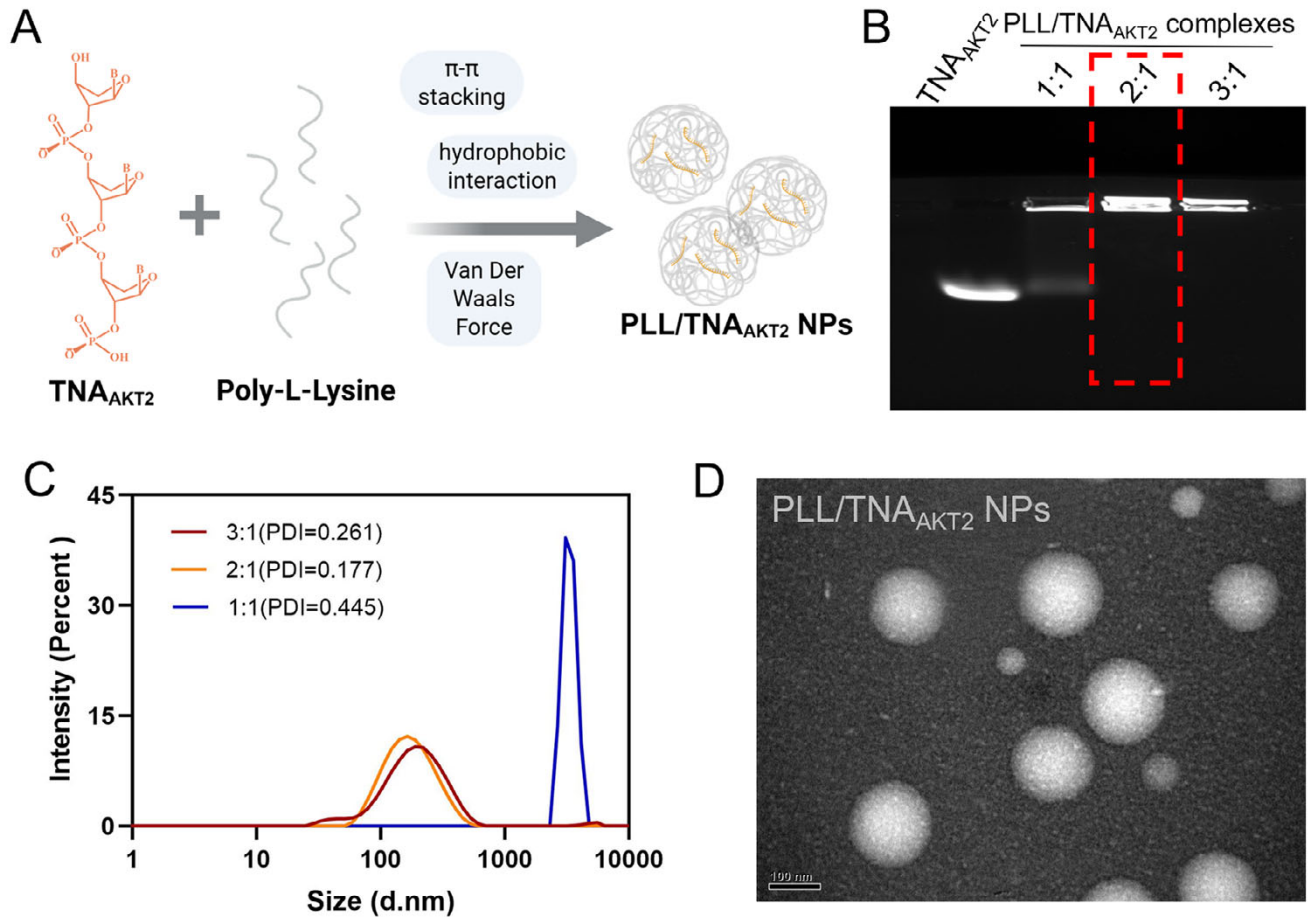
Characterization and optimization of PLL/TNA_AKT2_ core nanoparticles. (A) Schematic illustration of PLL/TNA_AKT2_ nanoparticle self‐assembly via electrostatic interactions. (B) Agarose gel retardation assay of PLL/TNA_AKT2_ complexes at various N/P ratios, showing complete TNA condensation at ratios ≥ 2:1. (C) Hydrodynamic diameter and PDI of PLL/TNA_AKT2_ NPs measured by DLS, identifying N/P 2:1 as the optimal formulation. (D) Representative TEM image of PLL/TNA_AKT2_ NPs at N/P 2:1, revealing a uniform spherical morphology. Scale bar: 100 nm.

The therapeutic core of the biomimetic nanoplatform was engineered via the electrostatic self‐assembly of cationic polymer poly‐L‐lysine (PLL) and anionic TNA‐based antisense oligonucleotides (TNA_AKT2_), as illustrated in Figure 1A. The TNA_AKT2_ sequences, designed for high‐affinity mRNA binding, were synthesized using automated solid‐phase phosphoramidite chemistry. Their molecular weights were validated by matrix‐assisted laser desorption/ionization time‐of‐flight (MALDI‐TOF) mass spectrometry (Figure S1). To optimize the nano‐architecture for subsequent bio‐interfacial cloaking, PLL/TNA_AKT2_ complexes were formulated across a range of nitrogen‐to‐phosphorus (N/P) ratios. Agarose gel electrophoresis revealed that TNA_AKT2_ was quantitatively condensed at N/P ratios ≥ 2:1, with complete retention of TNA within the loading wells (Figure 1B and Figure S2). Dynamic light scattering (DLS) analysis identified the N/P 2:1 ratio as the optimal formulation, yielding a hydrodynamic diameter of 122.4 ± 21.12 nm and highly monodisperse population (PDI = 0.177) (Figure 1C). This monodisperse population is critical for ensuing uniform coating and predictable in vivo pharmacokinetics. In contrast, formulations with an N/P ratio of 1:1 resulted in substantially larger hydrodynamic diameters and a high PDI of 0.445. This increase is likely attributable to incomplete charge neutralization at suboptimal PLL concentrations, leading to the formation of loosely packed or aggregated complexes through interparticle bridging by excess free TNA. Considering the high charge density and adhesive properties inherent to cationic PLL, the minimum N/P ratio required for quantitative TNA condensation was prioritized to optimize both colloidal stability and biocompatibility. This streamlined formulation strategy minimizes potential cytotoxicity and ensures a well‐defined substrate for high‐fidelity biomimetic functionalization. Consequently, an N/P ratio of 2:1 was established as the optimal stoichiometric balance and was standardized for all subsequent investigations. Transmission electron microscopy (TEM) revealed discrete, spherical nanostructures (Figure 1D and Figure S3) with a mean dry‐state diameter of 77.34 ± 25.09 nm (Figure S4). It is noted that the ± 25 nm variability which represents the standard derivation (SD) of individual diameters measured manually across multiple micrographs, is inherently higher in TEM due to the “dry‐state” collapse of the membrane shell and the stochastic nature of manual particle counting. Conversely, DLS provides a robust, ensemble‐averaged measurement of the hydrodynamic volume of thousands of particles in a hydrated, native state. Therefore, the characteristic discrepancy between the DLS‐measured hydrodynamic size and the TEM‐derived diameter is expected and understandable which is possibly. As shown in Figure S4B, a statistical analysis of >100 individual nanoparticles reveal a tight normal (Gaussian) distribution centered at 77.34 nm. This confirms the population is unimodal and lacks “outlier” clusters or significant heterogeneity. This minor size dispersity is a natural consequence of using PLL, which is a polydisperse cationic polymer (MW range 150 000–300 000 Da). The chain length distribution of PLL introduces subtle variations in the degree of nucleic acid condensation, yet the consistent bulk PDI value < 0.3 measured by DLS confirms that these variations do not compromise batch‐to‐batch reproducibility or colloidal stability. Collectively, these results confirm the successful assembly of stable, well‐defined PLL/TNA_AKT2_ core nanoparticles, providing a robust foundation for biomimetic functionalization.

### 2.2 Biomimetic Cloaking with TNBC Cell‐Derived Membranes

**FIGURE 2:**
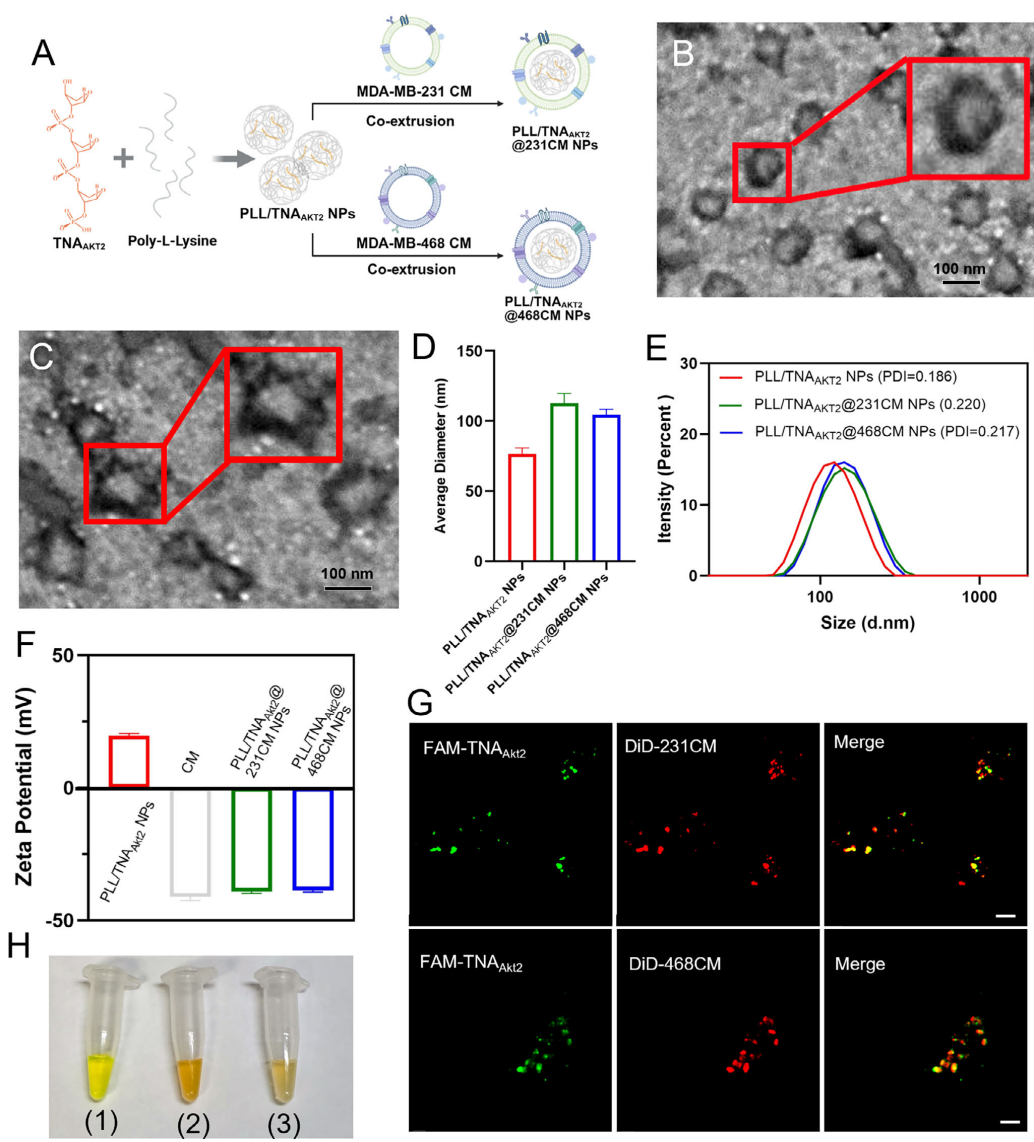
Synthesis and verification of biomimetic PLL/TNA_AKT2_@CM nanoparticles. (A) Schematic illustration of the preparation process for PLL/TNA_AKT2_@231CM and PLL/TNA _AKT2_@468CM NPs via mechanical extrusion. TEM images of (B) PLL/TNA _AKT2_@468CM NPs and (C) PLL/TNA _AKT2_@231CM NPs with negative staining. The distinct gray shell (arrowheads) confirms the presence of the lipid bilayer. Scale bars: 100 nm. (D) Statistical analysis of nanoparticle core and core–shell diameters from TEM images, confirming successful membrane coating. (E) DLS size distribution profiles showing the increased hydrodynamic diameter of nanoparticles after membrane coating. (F) Zeta potential measurements, indicating a charge reversal from positive (+19.3 mV) for uncoated NPs to negative (≈ −38 mV) for membrane‐coated NPs. (G) Confocal microscopy images showing high colocalization (Pearson's coefficient > 0.8) of DiD‐labeled membranes (red) and FAM‐labeled TNA cores (green) within the coated NPs. Scale bar: 5 µm. (H) Digital photographs of sample solutions, showing a distinct color change from bright yellow (free TNA) to light orange after membrane coating.

To impart homotypic targeting capabilities and biological functionality, the pre‐formed PLL/TNA_AKT2_ core NPs were encapsulated within plasma membranes derived from specific TNBC cell lines (MDA‐MB‐468 or MDA‐MB‐231) via a standardized mechanical co‐extrusion process (Figure 2A). The successful coating of the biomimetic nanoparticles was rigorously validated through multiple techniques. Direct visualization via TEM of PLL/TNA_AKT2_@468CM NPs (Figure 2B) and PLL/TNA_AKT2_@231CM NPs (Figure 2C) reveals a discrete, uniform, and continuous electron‐opaque shell surrounding the spherical polymeric cores. This characteristic core–shell architecture, indicative of a successfully translocated lipid bilayer, was distinctly absent in the uncoated PLL/TNA_AKT2_ controls. Similar structural motifs were observed in empty cell membrane vesicles (Figure S5), confirming that the contrasting peripheral layer originates from the biological membrane. Statistical analysis of the TEM images demonstrated that the mean diameter of the PLL/TNA_AKT2_@468CM NPs and PLL/TNA_AKT2_@231CM NPs increased to 104.52 ± 22.12 and 112.68 ± 27.05 nm, respectively (Figure 2D). This corresponds to an average membrane thickness of approximately 13.59 nm for the 468CM coating and 17.67 nm for the 231CM coating, consistent with the dimensions of a natural cellular bilayer. These findings were further corroborated by DLS, which showed a significant hydrodynamic size shift from 122.4±21.12 nm for the uncoated core to 135.2±20.15 nm (PDI: 0.217) and 141.8±22.40 nm (PDI: 0.220) for the respective membrane‐coated nanoparticles (Figure 2E). The final biomimetic formulations consistently exhibited PDI values below 0.3, which is the recognized threshold for monodisperse delivery systems in nanomedicine.

Furthermore, zeta potential measurements provided definitive evidence of complete membrane coating through a significant surface charge inversion. The highly cationic PLL/TNA _AKT2_ NPs of +19.3 mV underwent a dramatic reversal to a stable negative potential of approximately −38 mV upon coating (Figure 2F). This shift reflects the successfully shielding of the PLL core by the negative charged nucleic acids and phospholipids characteristic of the outward‐facing cell membrane glycocalyx. To verify the spatial integration of core and shell components, confocal laser scanning microscopy (CLSM) was employed using FAM‐labeled TNA cores (green) and DiD‐labeled membranes (red). The resulting fluorescence images showed high spatial colocalization of the green (FAM‐TNA_AKT2_) and red (DiD‐membrane) fluorescence signals, substantiated by a high Pearson's correlation coefficient (PCC > 0.8, Figure 2G). Notably, a discernible colorimetric transition was observed during discernible colorimetric transition fabrication process: the bright yellow of free FAM‐TNA_AKT2_ shifted to light orange upon PLL complexation, and finally to a pale orange following membrane coating (Figure 2H). This attenuation in fluorescence intensity is likely a result of the high‐density packing FAM fluorophores within the condensed core (self‐quenching) combined with the optical shielding effect provided by the biomimetic shell. Collectively, these multi‐modal characterization data confirm the successful fabrication of structurally intact, biomimetic PLL/TNA_AKT2_@CM NPs with preserved membrane architecture and high core–shell stability.

### 2.3 Retention of Proteomic Fidelity and Enhanced Colloidal Stability

**FIGURE 3:**
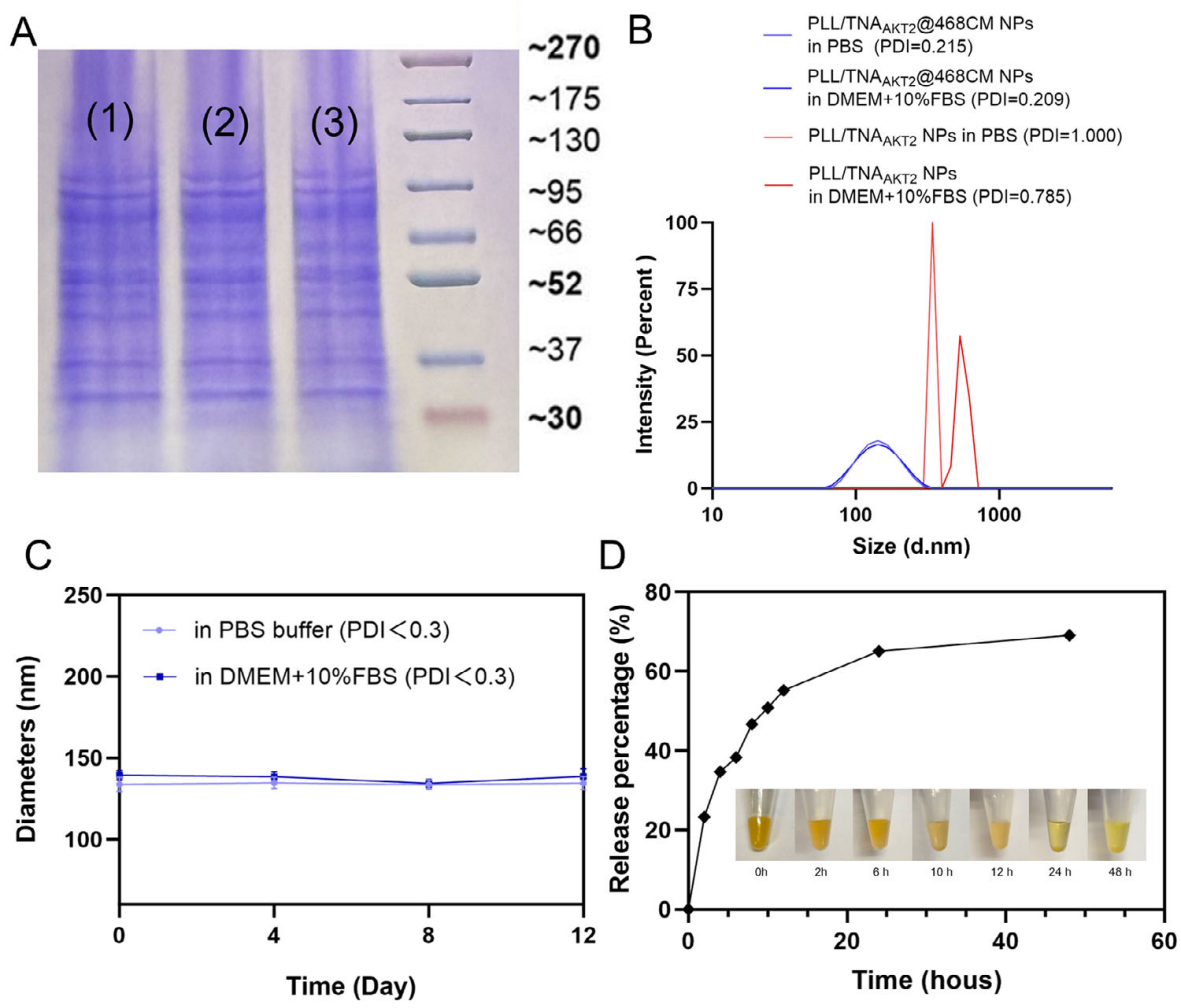
Enhanced biostability and controlled release of membrane‐coated nanoparticles. (A) SDS‐PAGE protein analysis confirming the preservation of the characteristic protein fingerprint and the relative abundance of major protein bands in PLL/TNA_AKT2_@468CM NPs after extrusion. Lane 1: Pure CM lysate; Lane 2: Purified cell membrane vesicles; and Lane 3: PLL/TNA_AKT2_@468CM NPs. (B) DLS analysis demonstrating the superior colloidal stability of PLL/TNA_AKT2_@468CM NPs compared to uncoated NPs, which aggregate severely in PBS and cell culture medium (DMEM + 10% FBS). (C) Long‐term stability of PLL/TNA_AKT2_@468CM NPs over 12 days, showing minimal change in hydrodynamic size and PDI. (D) In vitro release profile of TNA_AKT2_ from coated NPs under simulated intracellular conditions (pH 6.5, 0.1% trypsin), exhibiting a biphasic release pattern with a cumulative release of ∼70% over 48 h.

The preservation of the native membrane protein architecture is a prerequisite for maintaining the homotypic recognition and “biological stealth” capabilities of the nanoplatform. To evaluate the integrity of the membrane protein composition during the fabrication process, we performed SDS‐PAGE analysis on the source MDA‐MB‐468 cell membranes and the final PLL/TNA_AKT2_@468CM NPs (Figure 3A). The Coomassie‐stained gel revealed that the characteristic protein fingerprint and the relative abundance of major protein bands were highly conserved after extrusion. This demonstrates that the biomimetic coating process successfully retains the molecular complexity of the parent cell membrane, which is essential for preserving the homotypic targeting and fusion functionalities of the platform.

The biomimetic shell significantly enhanced the colloidal stability of the cationic cores under stimulated physiological conditions. The hydrodynamic diameter of PLL/TNA _AKT2_@468CM NPs in PBS buffer remained highly stable at 136.0 nm (DPI: 0.215), closely matching the dimensions observed in deionized water. In complex medium (DMEM supplemented with 10% FBS, the particle size increased only marginally to 140.43 nm with a PDI of 0.209, consistently below 0.3. This minimal shift indicates excellent resistance to non‐specific protein adsorption, likely due to the steric hindrance and electrostatic repulsion provided by the negatively charged, hydrophilic glycocalyx of the membrane shell. In contrast, uncoated PLL/TNA_AKT2_ NPs underwent immediate and irreversible aggregation upon exposure to electrolytes and serum proteins, with hydrodynamic diameters rapidly increasing to 342.00 nm (PBS) and 531.20 nm (medium), respectively (Figure 3B). Furthermore, long‐term stability assays demonstrated that the PLL/TNA_AKT2_@468CM NPs remained their monodisperse nature in both PBS and serum‐containing medium over 12 days of storage at 4°C. (Figure 3C). This long‐term colloidal stability in a protein‐rich environment suggests that the membrane coating is thermodynamically stable and effectively masks the PLL core under simulated physiological conditions.

Ensuring that the nucleic acid remains chemically intact during the mechanical and chemical stresses of nanoparticle fabrication is essential for maintaining therapeutic potency. We performed denaturing PAGE to monitor TNA integrity at each critical stage of the fabrication process. To rigorously verify that neither electrostatic complexation nor mechanical extrusion compromised the TNA backbone, we analyzed three specific samples including the native TNA_AKT2_ before any processing, TNA_AKT2_ incubated with the release agents (heparin and DMSO) used in the extraction protocol to ensure they do not induce degradation, and TNA_AKT2_ extracted from the final PLL/TNA_AKT2_@468CM NPs after the complete fabrication process (complexation followed by 11 passes of sequential extrusion). As shown in the Figure S6, all three samples exhibited a single, sharp band with identical electrophoretic mobility. There were no detectable truncated fragments, low‐molecular‐weight species, or “smearing” typically associated with backbone cleavage or chemical degradation. These results confirm that the TNA backbone remains fully intact during the mechanical shear of sequential extrusion through 200 nm pores.

The release profile of TNA_AKT2_ was evaluated under simulated intracellular conditions (pH 6.5, 0.1% trypsin, 37°C) to mimic the proteolytic and mildly acidic microenvironment. The release kinetics exhibited a biphasic pattern, with an initial burst release of approximately 35% within the first 6 h, followed by a controlled, sustained release reaching a cumulative total of 69.1% over 48 h (Figure 3D). This controlled release behavior is therapeutically advantageous; the initial phase provides rapid cytoplasmic availability of the TNA payload upon internalization, while the subsequent sustained phase ensures prolonged inhibition of the AKT2 pathway for durable gene silencing effects.

**FIGURE 4:**
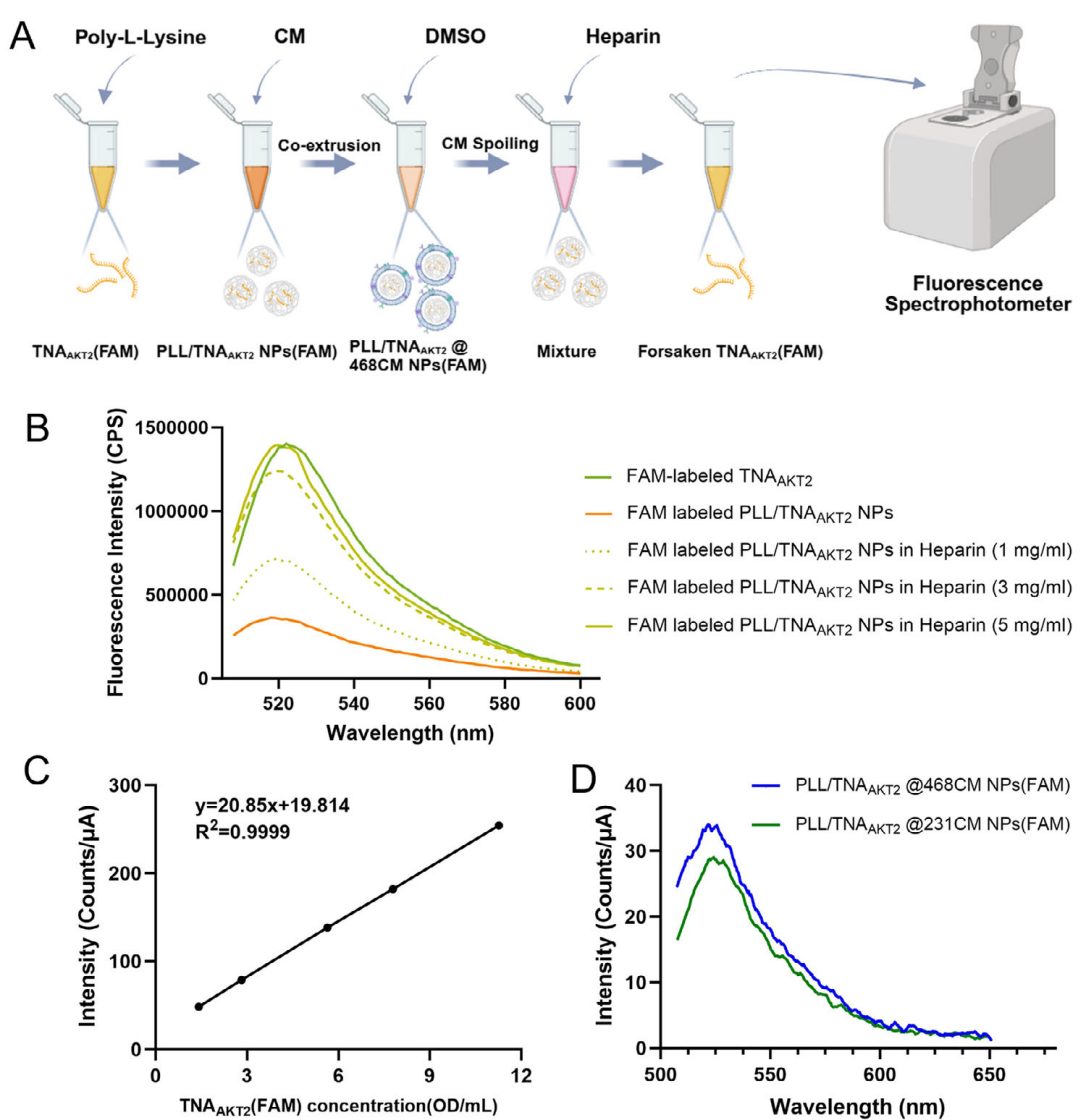
Quantification of TNA_AKT2_ loading efficiency in biomimetic nanoparticles. (A) Schematic illustration of the fluorescence‐based method for determining TNA_AKT2_ encapsulation efficiency. The cell membrane coating is disrupted with DMSO, and TNA_AKT2_ is subsequently displaced from the PLL carrier by heparin, resulting in the restoration of FAM fluorescence. (B) Fluorescence intensity recovery of FAM‐labeled TNA dissociated from PLL/TNA_AKT2_ NPs across various heparin concentration. Complete fluorescence restoration was achieved at a heparin concentration of 5 mg/mL. (C) The calibration curve for FAM‐labeled TNA_AKT2_ quantification showing linear relationship between FAM fluorescence intensity and TNA_AKT2_ concentration (R^2^ > 0.99), ensuring the high precision of our final process yield quantification. (D) The calculated final process yields of PLL/TNA_AKT2_@468CM NPs and PLL/TNA_AKT2_@231CM NPs, demonstrating efficient TNA loading of 36.3% and 30.2%, respectively.

The loading efficiency of the encapsulated TNA payload after membrane coating and mechanical extrusion was rigorously determined via a fluorescence quenching assay (Figure 4A,B). This method leverages the high‐density packing of FAM‐labeled TNA_AKT2_ within the PLL core, which results in significant self‐quenching of the fluorophores. To quantify the total entrapped cargo, the biomimetic shell was first solubilized using DMSO, followed by the introduction of heparin sodium—a highly anionic glycosaminoglycan. Heparin, possessing the highest charge density of any known biological macromolecule, acts as a competitive polyanion to displaces TNA from the cationic PLL backbone. To validate the 100% release threshold, we performed a heparin concentration gradient experiment (Figure 4C). At a concentration of 5 mg/mL heparin, FAM fluorescence intensity fully recovered to levels indistinguishable from the free FAM‐labeled TNA_AK2_TNA_FAM_ control, confirming quantitative displacement of TNA from the PLL matrix. Concentrations below 5 mg/mL resulted in incomplete recovery, indicating residual PLL‐TNA association. Therefore, 5 mg/mL heparin was used for all quantification experiments to ensure complete TNA release and accurate determination of final process yield. The subsequent restoration of FAM fluorescence was measured and calibrated against a standard curve generated by simulating total membrane disruption and heparin‐mediated release. As shown in Figure 4D, the loading efficiencies were calculated to be 36.3% for PLL/TNA_AKT2_@468CM NPs and 30.2% for PLL/TNA_AKT2_@231CM NPs. These values represent the overall process yield of the final biomimetic nanoplatform after membrane coating and mechanical extrusion, rather than the initial complexation efficiency of the cationic PLL/TNA core. Notably, this represents a significant loading capacity for genetic antisense oligonucleotides compared to comparable to or even exceed reported values for similar membrane‐cloaked nucleic acid systems (typically 18% to 36%) [50, 51, 52, 60]. This superior loading capacity is attributed to the strong electrostatic affinity between the PLL backbone and the TNA phosphate backbone, coupled with the effective “sealing” effect provided by the co‐extruded lipid bilayer, which minimizes cargo leakage during the fabrication process.

### 2.4 Homotypic Targeting Enables Preferential Uptake in Drug‐Resistant TNBC Cells

**FIGURE 5:**
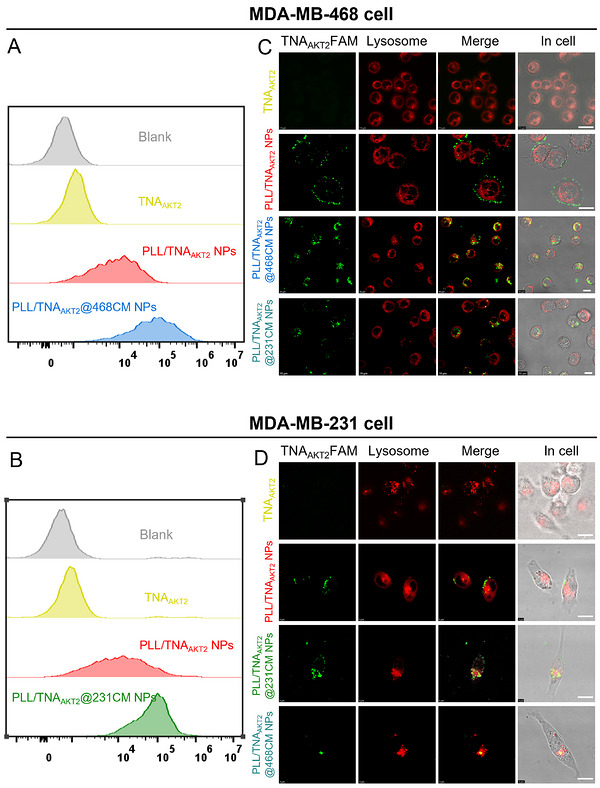
Homotypic targeting enables superior cellular uptake in drug‐resistant TNBC cells. FCM analysis of FAM‐labeled TNA uptake in (A) MDA‐MB‐468 and (B) MDA‐MB‐231 cells after 24 h treatment with various formulations. Homotypic membrane coating results in ∼100‐fold higher fluorescence intensity compared to free TNA. Confocal microscopy images confirming the efficient internalization of homotypic nanoparticles and their co‐localization with lysosomes in (C) MDA‐MB‐468 and (D) MDA‐MB‐231 cells. Uncoated NPs largely adhere to the cell surface. Scale bar: 7.5 µm.

The therapeutic efficacy of genetic medicines is fundamentally limited by their ability to achieve efficient intracellular delivery within specific cellular populations. We evaluated the cellular uptake kinetics and specificity of the biomimetic nanoplatform across two distinct TNBC molecular subtypes: MDA‐MB‐468 (drug‐resistant, Basal‐like 1) and MDA‐MB‐231 (Mesenchymal Stem‐like). Flow cytometry (FCM) was utilized to quantify the uptake of FAM‐labeled PLL/TNA_AKT2_@468CM NPs and PLL/TNA_AKT2_@231CM NPs. In both cell lines, incubation with homotypic cell membrane‐coated nanoparticles resulted in a profound shift in fluorescence intensity—exceeding a 100‐fold increase compared to cells treated with free TNA_AKT2_ (Figure 5A,B). While uncoated PLL/TNA_AKT2_ NPs also induced a moderate increase in signal, this is likely attributable to the high charge density of the cationic PLL core. Cationic particles frequently exhibit non‐specific electrostatic sequestration onto the negatively charged glycocalyx of the cell surface and culture substrate, which can lead to inflated fluorescence readings in FCM that do not accurately represent internalized cargo. To distinguish between surface‐adsorbed aggregates and true intracellular translocation, CLSM was performed (Figure 5C,D). CLSM images showed that uncoated NPs predominantly existed as extracellular clusters adhered to the plasma membrane, with negligible evidence of cytosolic penetration. In contrast, cells treated with their respective donor‐derived biomimetic nanoparticles exhibited diffuse, robust intracellular fluorescence. This confirms that the membrane coating does not merely increase the quantity of cell‐associated particles but fundamentally alters the internalization pathway, facilitating successful translocation into the cytoplasm.

The specificity of “biological cloak” was further validated through heterotypic cross‐pairing experiments. When drug‐resistant MDA‐MB‐468 cells were incubated with heterotypic PLL/TNA_AKT2_@231CM NPs, intracellular fluorescence was markedly attenuated compared to the homotypic combination. Similarly, PLL/TNA_AKT2_@468CM NPs failed to promote significant uptake in MDA‐MB‐231 cells.

To rigorously validate the subtype‐specific targeting and “intra‐disease” discrimination of PLL/TNA_AKT2_@468CM NPs, flow cytometry‐based uptake experiments comparing the donor‐matched MDA‐MB‐468 cells against two critical controls: MCF‐7/ADR (a doxorubicin‐resistant, non‐TNBC breast cancer), and MCF‐10A (normal mammary epithelial cells) were also conducted. We specifically selected MCF‐7/ADR as a non‐TNBC comparator because it shared a pronounced drug‐resistant phenotype with MDA‐MB‐468, allowing us to determine if homotypic recognition is driven by specific TNBC surface signatures or merely membrane features associated with chemoresistance. As shown in Figure S7, the PLL/TNA_AKT2_@468CM NPs exhibited preferential internalization by the donor‐matched MDA‐MB‐468 cells, while uptake was significantly attenuated in both the normal MCF‐10A cells and the drug‐resistant MCF‐7/ADR cells. These results confirm that our platform's homotypic affinity is dictated by specific TNBC subtype determinants rather than non‐specific tumor or resistance‐associated membrane interactions. These cellular uptake results demonstrate that membrane coating derived from a particular TNBC subtype preferentially facilitates uptake into the same cell type, a phenomenon likely mediated by homologous adhesion molecules and compatible membrane lipid composition. The resistance of MDA‐MB‐468 cells to heterotypic nanoparticles is particularly noteworthy, as it underscores the potential of this biomimetic platform to overcome the drug‐resistant phenotype that renders these cells refractory to conventional TNA delivery. Our findings establish that homotypic membrane functionalization is essential for achieving efficient subtypes‐selective internalization in TNBC, while uncoated cationic nanoparticles generate false‐positive signals in flow cytometry due to surface adhesion rather than true uptake. Our findings demonstrate that biomimetic membrane‐coated nanoparticles can be engineered to specifically target individual drug‐resistant TNBC subtypes by utilizing cell membranes derived from those certain subtypes, without the need for additional targeting ligands such as aptamers or peptides [49, 53]. This homotypic targeting capability paves the way for the development of highly customized nanomedicines that address the challenges of delivering TNA‐based drugs to drug‐resistant TNBC cells. Given that TNBC is a heterogeneous disease composed of multiple molecular subtypes that respond differently to treatment, our system's ability to discriminate and selectively target specific subtypes is crucial for maximizing therapeutic TNA efficacy while minimizing off‐target toxicity to non‐target TNBC subtypes. Therefore, our research establishes a framework for developing precision nanomedicine tailored to the unique surface markers of specific TNBC molecular subtypes.

### 2.5 Membrane Fusion‐Mediated Internalization Bypasses Endosomal Entrapment

**FIGURE 6:**
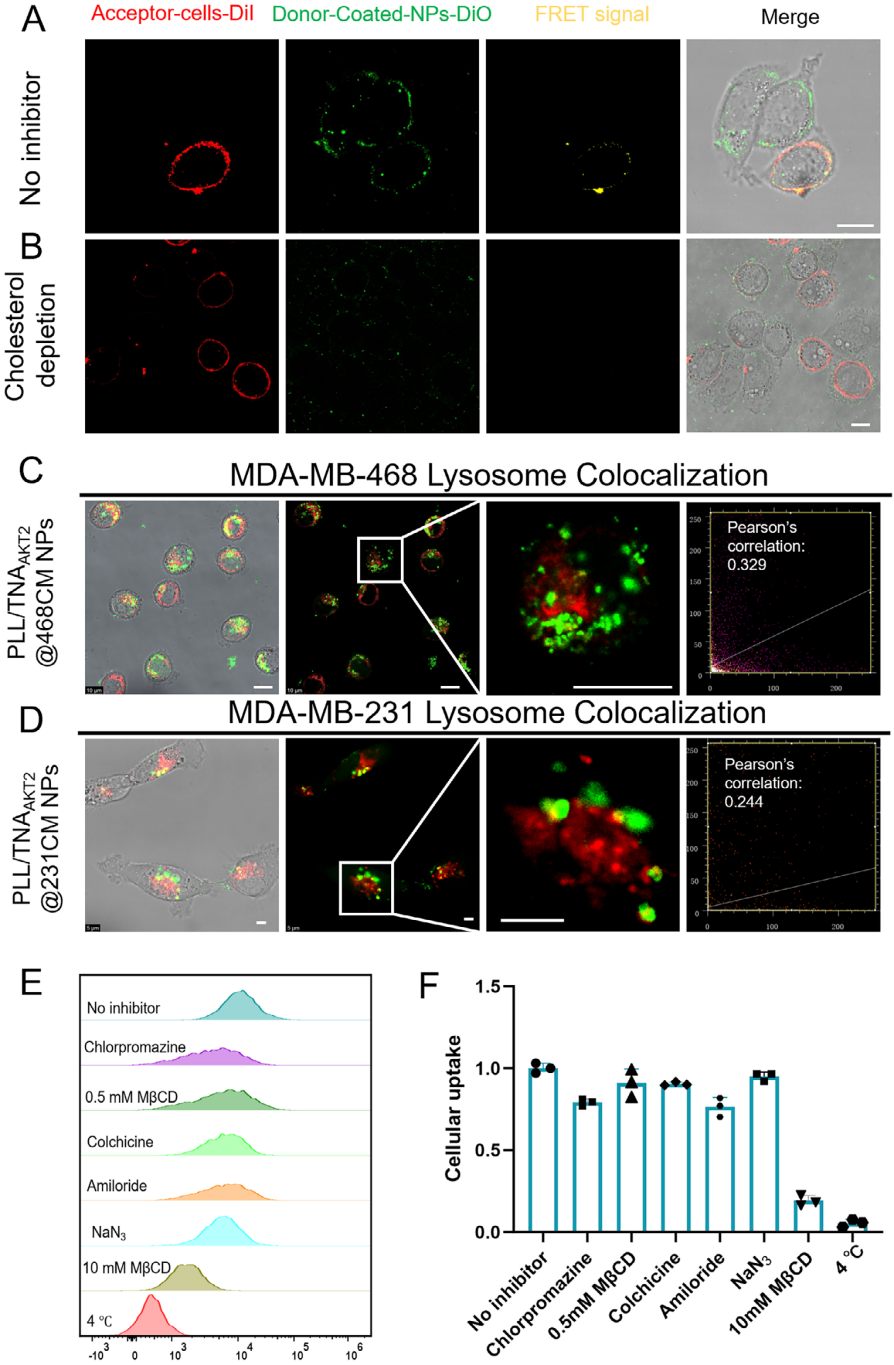
Membrane fusion‐mediated uptake facilitates efficient lysosomal escape. (A) Representative frames from a time‐lapse FRET‐based lipid mixing assay monitoring membrane fusion during cellular internalization. The biomimetic shell of the PLL/TNA_AKT2_@468CM NPs was labeled with DiO (donor), while the plasma membrane of live MDA‐MB‐468 cells was labeled with DiI (acceptor). Images were captured 4 min into a 5‐min recording period, initiating 55 min post‐incubation. Untreated MDA‐MB‐468 cells exhibiting a robust, sensitized DiI emission (FRET signal) at the cell periphery, confirming nanoparticles uptake via direct membrane fusion. (B) MDA‐MB‐468 cells pre‐treated with the cholesterol depleting agent methyl‐β‐cyclodextrin (MβCD), showing a markedly attenuated FRET signal consistent with inhibition of membrane fusion. Corresponding time‐lapse videos (in MP4 format) are provided in the revised (Videos S1–S4). Colocalization analysis of FAM‐TNA (green) with LysoTracker (red) in (C) MDA‐MB‐468 and (D) MDA‐MB‐231 cells treated with homotypic NPs. Low Pearson's coefficients (0.33 and 0.24) indicate efficient lysosomal escape. Scale bars: 10 µm. (E) FCM analysis of PLL/TNA_AKT2_@468CM NPs uptake in MDA‐MB‐468 cells following 24 h of treatment with various formulations. (F) Quantitative assessment of PLL/TNA_AKT2_@468CM NPs internalization in the presence of various pharmacological inhibitors. Cellular uptake was normalized to NP‐treated MDA‐MB‐468 cells in the absence of inhibitor.

A major bottleneck in genetic medicine is lysosomal sequestration, where therapeutic cargo is trapped and degraded within acidic lysosomes. While conventional vectors reply on endosomal escape, our biomimetic utilizes endosomal avoidance. To rigorously and visually distinguish between membrane fusion and rapid endosomal rupture, a real‐time Förster Resonance Energy Transfer (FRET)‐based lipid mixing assay was performed. The videos were recorded 4 min into a 5‐min recording period, initiated 55 min post‐incubation (Videos S1–S4). This assay provides high‐sensitivity evidence of the molecular proximity required for bilayer merging. In the experimental design, we utilized the lipophilic donor‐acceptor pair DiO (λ_ex_ = 488 nm, λ_em_ = 495–510 nm) and DiI (λ_ex_ = 543 nm, λ_em_ = 565–665 nm. The biomimetic MDA‐MB‐468 cell membrane shell of the PLL/TNA_AKT2_@468CM NPs was labeled with DiO. The plasma membrane of live MDA‐MB‐468 recipient cells was stained with DiI. Upon excitation at 488 nm, a FRET signal (sensitized emission of DiI) can only occur if the two lipid bilayers achieve molecular proximity (<10 nm), a state synonymous with lipid mixing during membrane fusion. As shown in Figure 6A and Videos S1 and S2, we observed a progressive increase in sensitized DiI emission FRET channel at the cell periphery within minutes of nanoparticle contact. The spatial distribution of the FRET signal initially restricted to the plasma membrane region, which is characteristic of direct fusion at the cell surface. This signal colocalized with the DiO‐labeled nanoparticles, indicating successful integration of the nanoparticle shell into the host cell plasma membrane. In cells lacking DiI staining, no FRET signal was detected despite nanoparticles binding, which exclude potential spectral bleed‐through artifacts. Furthermore, cells pre‐treated with Methyl‐β‐cyclodextrin (MβCD) to deplete membrane cholesterol, thereby reducing membrane fluidity and fusion potential, exhibited negligible FRET signals (Figure 6B and Videos S3 and S4). This FRET‐based lipid mixing assay provides direct, real‐time evidence of lipid bilayer fusion between the nanoparticle shell and the host cell plasma membrane. Nanoparticles internalized via endocytosis are typically confined within intracellular vesicles, such as endosomes, phagosomes, or macropinosomes, and therefore the nanoparticle membrane and the cellular plasma membrane remain physically separated by an intervening vesicle lumen. This spatial segregation precludes the molecular proximity (typically <10 nm) required for FRET [61]. In contrast, a robust and immediate FRET signal is observed at the cell surface only when the nanoparticle shell directly fuses with the plasma membrane. This event is a distinct mechanism by which lipid bilayer‐coated nanoparticles merge with the cell membrane and deliver their contents directly to the cytoplasm. Thus, this FRET‐based assay effectively differentiates membrane fusion from endocytic uptake. To further elucidate the intracellular fate of the nanoplatform, confocal fluorescence colocalization analysis between PLL/TNA_AKT2_@468CM NPs and LysoTracker Deep Red was performed. MDA‐MB‐468 and MDA‐MB‐231 cells treated with their respective homotypic FAM‐labeled TNA loaded nanoparticles exhibited minimal fluorescence overlap with the lysosomal compartment, yielding low Pearson's correlation coefficients of 0.329 and 0.244, respectively (Figure 6C,D). This lack of lysosomal colocalization confirms that the fusion‐mediated pathway bypasses the endolysosomal route entirely, transitioning the delivery paradigm from endosomal escape to endosomal avoidance.

To further define the internalization pathway, we performed pharmacological inhibitor assays and temperature‐dependent uptake studies quantified by FCM (Figure 6E,F). Chlorpromazine (clathrin‐mediated endocytosis inhibitor), colchicine (macropinocytosis inhibitor), amiloride (clathrin/caveolae‐mediated endocytosis inhibitor), low‐dose MβCD (caveolae‐mediated endocytosis inhibitor) did not significantly attenuate nanoparticle uptake, effectively ruling out macropinocytosis and clathrin/caveolae‐mediated endocytosis as primary internalization routes. In contrast, high‐dose MβCD treatment resulted in a profound reduction in cellular uptake (> 70% inhibition). We employed MβCD at two distinct concentrations to differentiate its effects on endocytosis versus membrane fusion [62]. Low‐dose MβCD (≤1 mm) was used to selectively disrupt cholesterol‐rich lipid rafts, thereby inhibiting caveolae‐ and clathrin‐mediated endocytosis without causing significant cytotoxicity. In contrast, high‐dose MβCD (10 mm) was used to more extensively deplete membrane cholesterol, which increase membrane bending rigidity and reduced lateral mobility to levels that physically block the lipid mixing and stalk formation required for membrane fusion.

Additionally, the near‐complete attenuation of nanoparticle uptake at 4°C is primarily attributed to the thermotropic phase transition of the plasma from a fluid, liquid‐crystalline state to a rigid gel phase [63]. This transition significantly increases the membrane's bending modulus and reduces lateral lipid mobility, thereby creating a physical barrier to the structural rearrangements required for fusion, such as stalk formation and pore expansion. In contrast, our results demonstrated that uptake remains largely operational under NaN_3_ treatment at 37°C. While NaN_3‐_indcued ATP depletion halts energy‐dependent processes like clathrin‐mediated scission and actin‐driven macropinocytosis, the biophysical merger of lipid bilayer merger is a thermodynamically spontaneous process once membranes are in close apposition [64]. This comprehensive pharmacological profile, combined with the FRET‐based lipid mixing data, provides robust and multi‐faceted evidence that PLL/TNA_AKT2_@468CM NPs internalize predominantly via a non‐canonical, cholesterol‐dependent membrane fusion pathway rather than conventional endocytic mechanisms.

Although PLL/TNA_AKT2_@CM NPs avoid retention in the acidic organelles, the release rate of therapeutic nucleic acids will not be reduced because the release of TNA from our platform is primarily governed by the proteolytic degradation of the PLL carrier, rather than a strict requirement for acidic pH. PLL is a synthetic polypeptide susceptible to a wide array of proteases, including cathepsins, trypsin‐like enzymes, and various cytosolic peptidases. Our in vitro release assay (pH 6.5 + trypsin) was designed to mimic the tumor microenvironment (TME). The TME is characterized by mild acidity (pH 6.5–6.8) and high extracellular protease activity. This assay demonstrates that the system is “TME‐responsive,” ensuring that even nanoparticles that do not immediately fuse can begin releasing their cargo in the peritumoral space. Once the PLL/TNA core is delivered directly into the cytosol via membrane fusion, it is exposed to cytosolic proteases. While the cytosolic pH is relatively neutral (∼7.2), many intracellular proteases and the proteasomal machinery remain highly active at this pH. Consequently, PLL degradation and subsequent TNA release proceed efficiently in the cytoplasm, independent of endosomal acidification.

By ensuring direct translocation into the cytosol, the PLL/TNA_AKT2_@CM system maximizes the bioavailability of the genetic cargo for target mRNA engagement. While TNA oligonucleotides are uniquely characterized by their inherent resistance to nuclease‐mediated degradation, the protection and direct‐entry mechanism afforded by this biomimetic shell have broader implications for the field of nanomedicine. The ability to circumvent the harsh, enzymatic environment of the lysosome suggests that this platform can be readily adapted to safeguard and deliver a wide spectrum of more labile therapeutic payloads such as mRNA, siRNA, or CRISPR/Cas9 ribonucleoproteins which are typically inactivated by lysosomal acidification and proteolysis. Consequently, these findings establish membrane‐fusion delivery as a superior and highly versatile strategy for genetic therapeutics, providing an elegant, high‐efficiency solution to the endosomal sequestration barrier that has long hindered the clinical translation of advanced biologics.

### 2.6 Potent AKT2 Silencing and Targeted Antitumor Activity in Vitro

**FIGURE 7:**
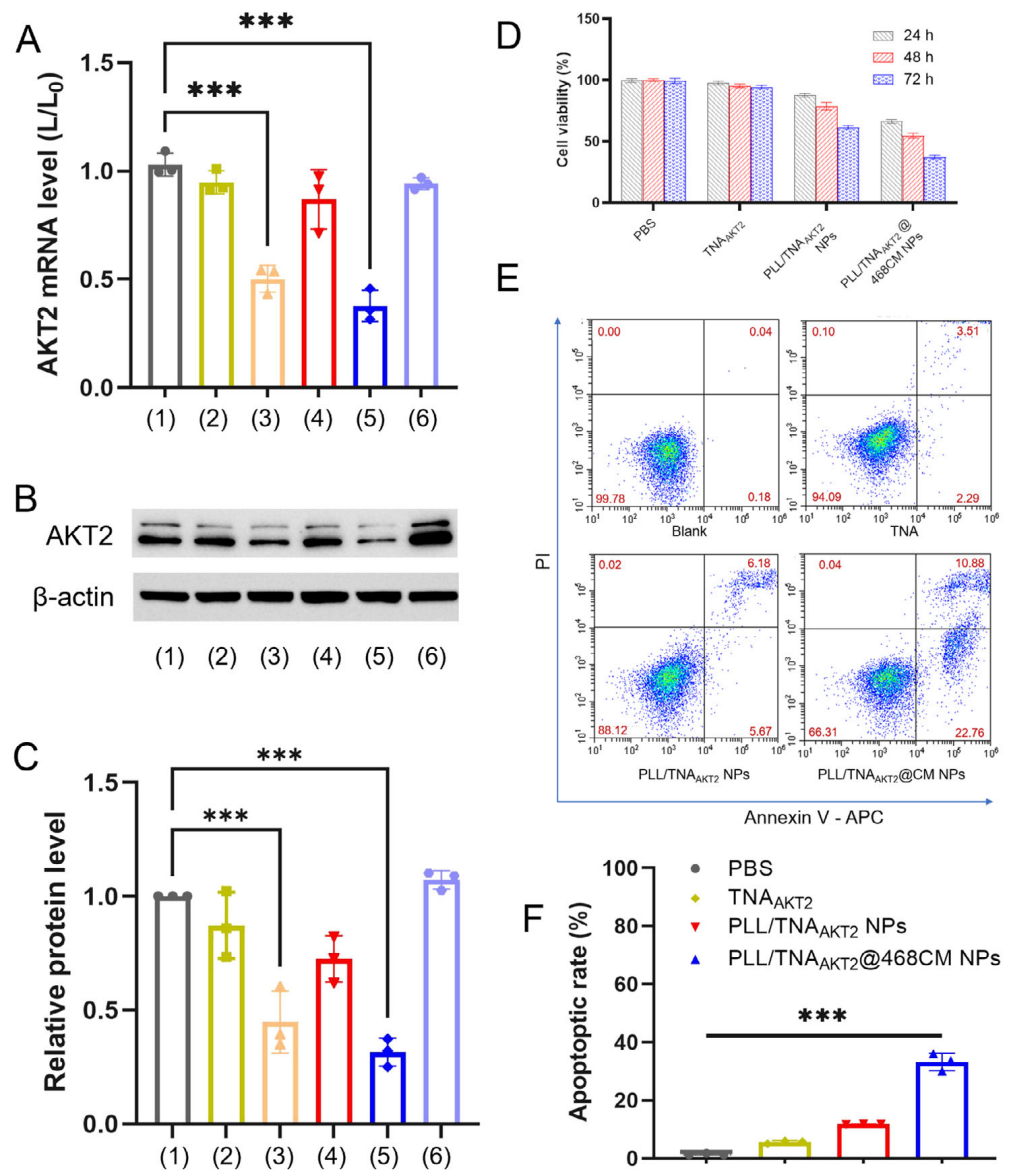
Potent and specific AKT2 gene silencing by PLL/TNA_AKT2_@468CM NPs in vitro. (A) Relative AKT2 mRNA expression in MDA‐MB‐468 cells after 48 h treatment, measured by RT‐qPCR. (B) Western blot analysis and (C) quantification of AKT2 protein levels after 72 h treatment. (1) Blank, (2) TNA_AKT2_, (3) Lipo3000 transfected‐TNA_AKT2_, (4) PLL/TNA_AKT2_ NPs, (5) PLL/TNA_AKT2_@468CM NPs, and (6) PLL/ TNA_SCR_@468CM NPs. (D) Cell viability (MTT assay) of MDA‐MB‐468 cells over 72 h, demonstrating the superior anti‐proliferative effect of PLL/TNA_AKT2_@468CM NPs. (E) FCM analysis of Annexin V‐APC/PI‐stained cells and (F) quantification of apoptotic rates after 48 h treatment. Data are presented as mean ± SD (n = 3). ^*^ *p* < 0.05, ^**^ *p* < 0.01, ^***^ *p* < 0.001.

By leveraging homotypic targeting and membrane fusion‐mediated internalization, the PLL/TNA_AKT2_@468CM NPs were evaluated for their capacity to silence the oncogenic driver AKT2 and suppress the proliferation of drug‐resistant MDA‐MB‐468 cells. Given its role as a master regulator of survival in this TNBC subtype, AKT2 represents a critical therapeutic vulnerability. Gene silencing efficacy was first quantified at the mRNA level via RT‐qPCR following 48 h of treatment (Figure 7A). Consistent with their poor cellular internalization, neither free TNA_AKT2_ nor uncoated PLL/TNA_AKT2_ NPs elicited significant knockdown. In contrast, PLL/TNA_AKT2_@468CM NPs reduced AKT2 mRNA expression by 63.0% relative to the control. This results substantially outperformed the gold‐standard Lipofectamine 3000‐mediated transfection, which achieved 50.0% knockdown under identical conditions. The absence of silencing in cells with scrambled TNA (TNA_SCR_)‐loaded coated NPs confirmed that the observed effect was sequence‐specific. Western blot analysis corroborated these findings at the translational level (Figure 7B,C). After 72 h, PLL/TNA_AKT2_@468CM NPs reduced AKT2 protein expression to 31.5% of baseline levels, whereas Lipofectamine‐transfected TNA_AKT2_ only achieved a reduction to 44.7%. Uncoated NPs and free TNA_AKT2_ failed to induce appreciable protein depletion. The potent AKT2 silencing observed at both the mRNA and protein serves as definitive evidence that the TNA payload is successfully liberated from the PLL core in the cytosol and the release kinetics are sufficient to allow the TNA to inhibit the expression of target mRNAs. Furthermore, the intrinsic chemical stability of TNA is a key advantage in this study. Unlike siRNA, TNA's threose backbone is highly resistant to nucleases, ensuring that it remains functional during the window of time required for the PLL carrier to degrade in the neutral cytosolic environment. Importantly our platform's silencing efficiency markedly exceeds previously reported by Qiu et al., highlighting the functional superiority of TNA‐based antisense agents when coupled with efficient cytosolic delivery [54].

The requirement for homotypic recognition was further reinforced by heterotypic pairing. PLL/TNA_AKT2_@231CM NPs failed to induce significant knockdown in MDA‐MB‐468 cells (Figure S8). This emphasizes that the “like‐targets‐like” interaction is a prerequisite for the functional translocation of the TNA payload into the cytosol. Collectively, these findings demonstrate that cell membrane‐coated nanoparticles enable effective delivery of therapeutic agents into drug‐resistant TNBC cells, achieving potent silencing of the target gene at both mRNA and protein levels. This strategy successfully overcomes the previously reported challenge of poor TNA uptake in resistant TNBC subtypes, establishing a viable approach for antisense therapy in refractory cancers [42].

To assess the phenotypic impact of AKT2 depletion which is known to disrupt constitutive PI3K/AKT survival signaling [65], we performed cell viability and apoptosis assays. Methyl tetrazolium (MTT) assays revealed that PLL/TNA_AKT2_@468CM NPs progressively inhibited MDA‐MB‐468 proliferation over 72 h, reducing viability to 37.3%. This was significantly more potent than uncaoted PLL/TNA_AKT2_ NPs (65.2%) or free TNA_AKT2_ (>95%) (Figure 7D). The inherent biocompatibility of the PLL carrier was confirmed independently, with cell viability remaining above 95% even at concentrations up to 100 nm (Figure S9). Apoptosis induction was quantified via Annexin V‐APC/PI (Figure 7E,F). PLL/TNA_AKT2_@468CM NPs triggered apoptosis in 33.2% of cells population, a marked increase over uncoated NPs (11.8%) and free TNA_AKT2_ (2.0%). This robust pro‐apoptotic response is a direct consequence of the platform's ability to bypass endosomal entrapment and delivery TNA_AKT2_ directly to its cytosolic target, effectively abrogating the pro‐survival AKT2 signaling cascade. Collectively, the synergistic integration of homotypic targeting, membrane fusion‐mediated endosomal avoidance, and the intrinsic biostability of TNA enables the potent and specific reversal of the drug‐resistant TNBC phenotype. This positions our biomimetic platform as a high‐fidelity strategy for nucleic acid therapy in refractory cancers.

#### 2.6.1 In Vivo Biodistribution and Tumor‐Specific Accumulation

**FIGURE 8:**
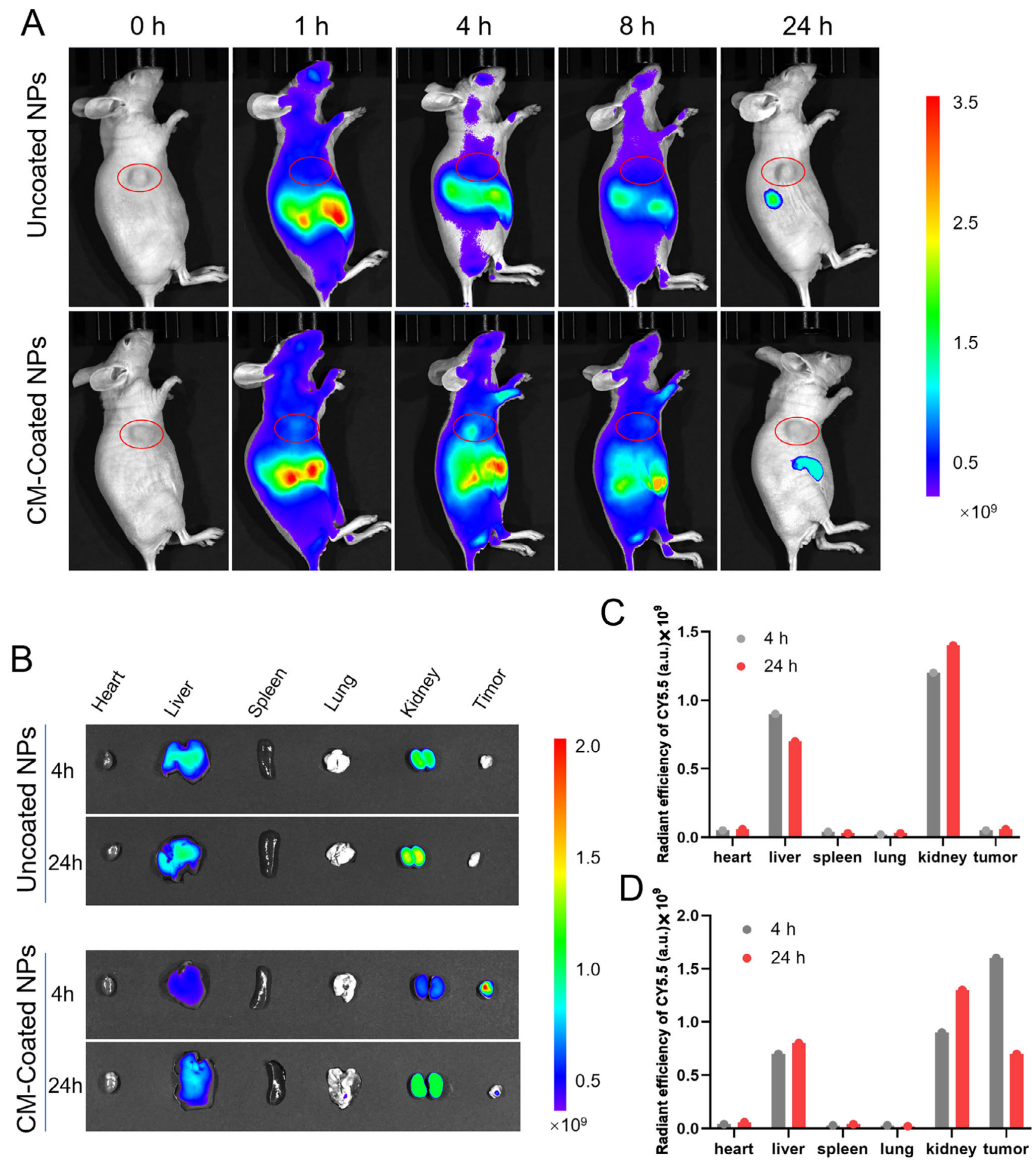
In vivo tumor targeting and biodistribution of PLL/TNA_AKT2_@468CM NPs (A) In vivo fluorescence imaging of MDA‐MB‐468 tumor‐bearing mice at indicated time points after intravenous injection of PBS, PLL/Cy5.5‐TNA_AKT2_ NPs, or PLL/Cy5.5‐TNA_AKT2_@468CM NPs. Tumors are circled in red. (B) Ex vivo fluorescence images of major organs and tumors harvested at 4 and 24 h post‐injection. Quantitative analysis of Cy5.5 fluorescence signal in organs and tumors for uncoated (C) and coated (D) NPs, confirming the significantly enhanced and prolonged tumor accumulation of the biomimetic formulation.

To evaluate whether the biomimetic MDA‐MB‐468 membrane coating enhances systemic circulation and tumor‐specific accumulation, in vivo fluorescence imaging was performed on MDA‐MB‐468 tumor‐bearing BALB/c nude mice. Once tumors reached approximately 100 mm^3^, mice were intravenously administered either PLL/Cy5.5‐TNA_AKT2_ NPs (Uncoated NPs) or PLL/Cy5.5‐TNA_AKT2_@468CM NPs (CM‐coated NPs). The real‐time biodistribution and tumor accumulation were monitored via a whole‐body imaging system over 24 h (Figure 8A). Distinctly different pharmacokinetic profiles were observed between the two formulations. The uncoated NPs exhibited rapid systemic distribution with negligible tumor accumulation at all examined time points. By 4 h post‐injection, fluorescence signals were diffusely distributed throughout the body with no focal enrichment at tumor sites, and by 24 h, these signals were nearly undetectable, suggesting rapid systemic clearance and lack of tumor retention. In contrast, mice treated with CM‐coated NPs displayed strong fluorescence signals that localized specifically within the tumor region as early as 4 h post‐injection. This selective extravasation and retention demonstrate the potent tumor‐targeting capability conferred by the biomimetic membrane. The “biological cloak” likely provides a dual advantage: extending blood circulation time by evading the mononuclear phagocyte system (MPS) and facilitating high‐affinity interactions with the parent tumor cells. To quantitatively assess tissue distribution, major organs (heart, liver, spleen, lungs, kidneys) and tumors were harvested at 4 and 24 h post‐injection for ex vivo fluorescence imaging (Figure 8B) and subsequent quantitative analysis (Figure 8C,D). At 4 h, the CM‐Coated NPs group exhibited significantly higher fluorescence intensity within the tumor tissue compared to the Uncoated NP group. Remarkably, by 24 h, the tumor‐associated signal in the CM‐coated NPs group remained >10‐fold higher than that in the uncoated NP group, confirming superior tumor retention and eliminating membrane shedding. The stark contrast in accumulation levels strongly indicates that the homotypic targeting moieties remained functional throughout the circulation period. Both formulations showed anticipated accumulation in the liver and kidneys, reflecting standard hepatobiliary and renal clearance routes for nanomedicines. However, only the biomimetic formulation achieved meaningful, sustained tumor localization. These findings underscore the critical role of the cancer cell‐derived coating in dictating nanoparticle biodistribution and overcoming the biological barriers to systemic delivery. Such tumor‐specific accumulation provides a rigorous foundation for achieving in vivo therapeutic efficacy while minimizing off‐target exposure and associated systemic toxicity.

### 2.7 In Vivo Therapeutic Efficacy and Molecular Mechanism in Drug‐Resistant TNBC Xenografts

**FIGURE 9:**
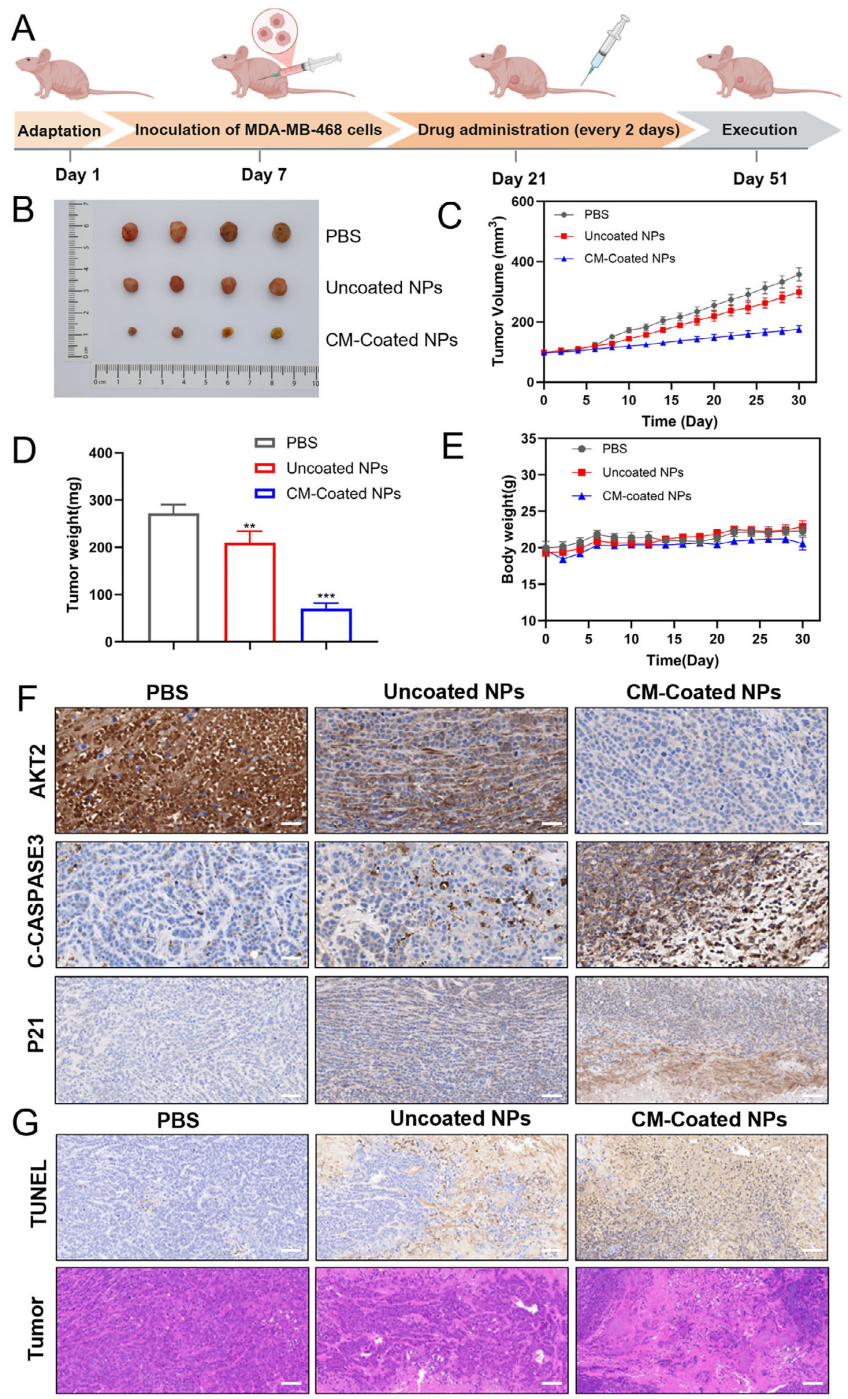
In vivo antitumor efficacy of PLL/TNA_AKT2_@468CM NPs in a drug‐resistant TNBC xenograft model. (A) Schematic representation of the treatment regimen. (B) Photographs of excised tumors from each group at the end of the study (Day 30). (C) Individual and mean tumor growth curves, showing significant suppression by PLL/TNA_AKT2_@468CM NPs. (D) Average tumor weights at Day 30. (E) Body weight changes of mice during treatment, indicating good tolerability. (F) Immunohistochemistry (IHC) staining of tumor sections for AKT2, p21, and cleaved Caspase‐3. The PLL/TNA_AKT2_@468CM NP group shows marked reduction in AKT2 and concurrent upregulation of p21 and cleaved Caspase‐3, confirming on‐target activity and induction of apoptosis. (G) TUNEL staining (top panel) of tumor sections, confirming extensive DNA fragmentation (apoptosis) in the treatment group. H&E staining (bottom panel) of tumor sections, revealing significant tumor tissue necrosis in the PLL/TNA_AKT2_@468CM NP group. Scale bars: 50 µm. Data are mean ± SEM (n = 4). ^*^ *p* < 0.05, ^**^ *p* < 0.01, ^***^ *p* < 0.001.

Building upon the superior tumor‐specific accumulation and endosomal avoidance capabilities observed, we evaluated the therapeutic potential of the PLL/TNA_AKT2_@468CM NPs in a drug‐resistant MDA‐MB‐468 xenograft mouse model. Once tumors reached a mean volume of approximately 100 mm^3^, mice were randomly assigned to three groups (n = 4 per group): PBS, uncoated PLL/TNA_AKT2_ NPs (Uncoated NPs), and PLL/TNA_AKT2_@468CM NPs (CM‐coated NPs). PBS serves as vehicle control to establish the baseline aggressive growth kinetics of the MDA‐MB‐468 xenograft model. Uncoated NPs served as a non‐targeted control to isolate the specific contribution of the biomimetic coating. Treatments were administered via tail vein injection every three days for a duration of 30 days, with longitudinal monitoring of tumor kinetics and systemic health (Figure 9A and Figure S10). The CM‐coated NPs demonstrated potent tumor growth inhibition compared to the control groups (Figure 9B,C). Tumors in the PBS and uncoated NP groups exhibited rapid and aggressive growth, with mean tumor volumes increasing 4.5 and 3.8‐fold, respectively, by the study endpoint. In contrast, the CM‐Coated NPs group showed substantial growth suppression of tumor progression, with volumes increasing only 1.8‐fold. This therapeutic efficacy was further validated by the final tumor weights, which were significantly lower in the CM‐Coated NP group compared to all controls (Figure 9D). Importantly, the stability of body weight across all treatment groups (Figure 9E) and the absence of behavioral changes suggest excellent systemic tolerability and a lack of overt acute toxicity. The differences between control groups and sample group demonstrated that the observed anti‐tumor activity is not an inherent property of the PLL/TNA core, but is strictly dependent on the homotypic targeting and fusion‐mediated entry provided by the membrane cloak.

To confirm that the observed antitumor effect was driven by sequence‐specific AKT2 knockdown, immunohistochemical (IHC) analysis was performed on the harvested tumor tissues (Figure 9F). Tumors from PBS and uncoated NP groups exhibited intense, widespread brown staining for AKT2, reflecting high protein expression. In contrast, the CM‐Coated NP‐treated tumors showed a markedly reduction in AKT2 immunoreactivity, providing definitive evidence of successful in vivo target engagement. We further investigated the downstream molecular consequences of AKT2 depletion. Consistent with the induction of a p21‐meidated apoptotic cascade, tumors from the CM‐Coated NP group displayed significantly upregulated expression of p21 expression and cleaved caspase‐3 (a hallmark of late‐stage apoptosis). Minimal immunoreactivity for these markers were detected in control groups, confirming that the observed cell death was a direct result of the TNA_AKT2_‐mediated disruption of pro‐survival signaling. These histological findings are consistent with previous studies using Tet‐on Akt shRNA models, reinforcing the validity of our approach. The overall significant tumor growth inhibition and robust AKT2 knockdown serve as functional proxies for clock retention. Our proposed mechanism relies on the membrane coating to facilitate fusion‐mediated cytosolic delivery. Without the integrity of the membrane cloak, the TNA_AKT2_ cargo would lack the “fusion‐gate” necessary to bypass endosomal entrapment and reach the cytosol. The successful suppression of the oncogenic driver in vivo presupposes that the membrane shell remained intact to engage with the target cell membrane.

The induction of programmed cell death within the tumor microenvironment was rigorously evaluated using the Terminal deoxynucleotidyl transferase dUTP nick end labeling (TUNEL) assay. Analysis of tumor sections revealed extensive DNA fragmentation in the CM‐Coated NP group, characterized by elevated brownish‐yellow particles indicative of apoptosis. In contrast, minimal apoptotic activity was detected in PBS and uncoated NP groups (Figure 9G, top panel), indicating that the therapeutic effect is specifically driven by the membrane‐targeted delivery of the TNA payload. These findings were further corroborated by hematoxylin and eosin (H&E) staining of the tumor tissues (Figure 9G, bottom panel). Collectively, these results demonstrate that systemic administration of CM‐Coated NPs achieves effective tumor targeting, robust in vivo gene silencing, and significant tumor growth inhibition in a drug‐resistant TNBC xenograft model, without observable toxicity. Since MDA‐MB‐468 cells are characterized by PTEN loss and EGFR amplification, leading to high PI3K/AKT pathway dependency and significant resistance to taxanes. Studies indicated that MDA‐MB‐468 cells exhibit an IC_50_ of ∼23 µm for docetaxel which is substantially higher than that of drug‐sensitive TNBC subtypes, confirming a significantly resistant phenotype [66]. Therefore, the biomimetic membrane coating is instrumental in overcoming the biological barriers to systemic delivery, enabling the stabilized TNA cargo to reach the cytosolic compartment and trigger the p21/caspase‐3 apoptotic pathway. This work established a viable and potent framework for antisense therapy in heterogeneous and drug‐resistant malignancies.

### 2.8 Biosafety Assessment of Biomimetic Nanoparticles in Vivo

**FIGURE 10:**
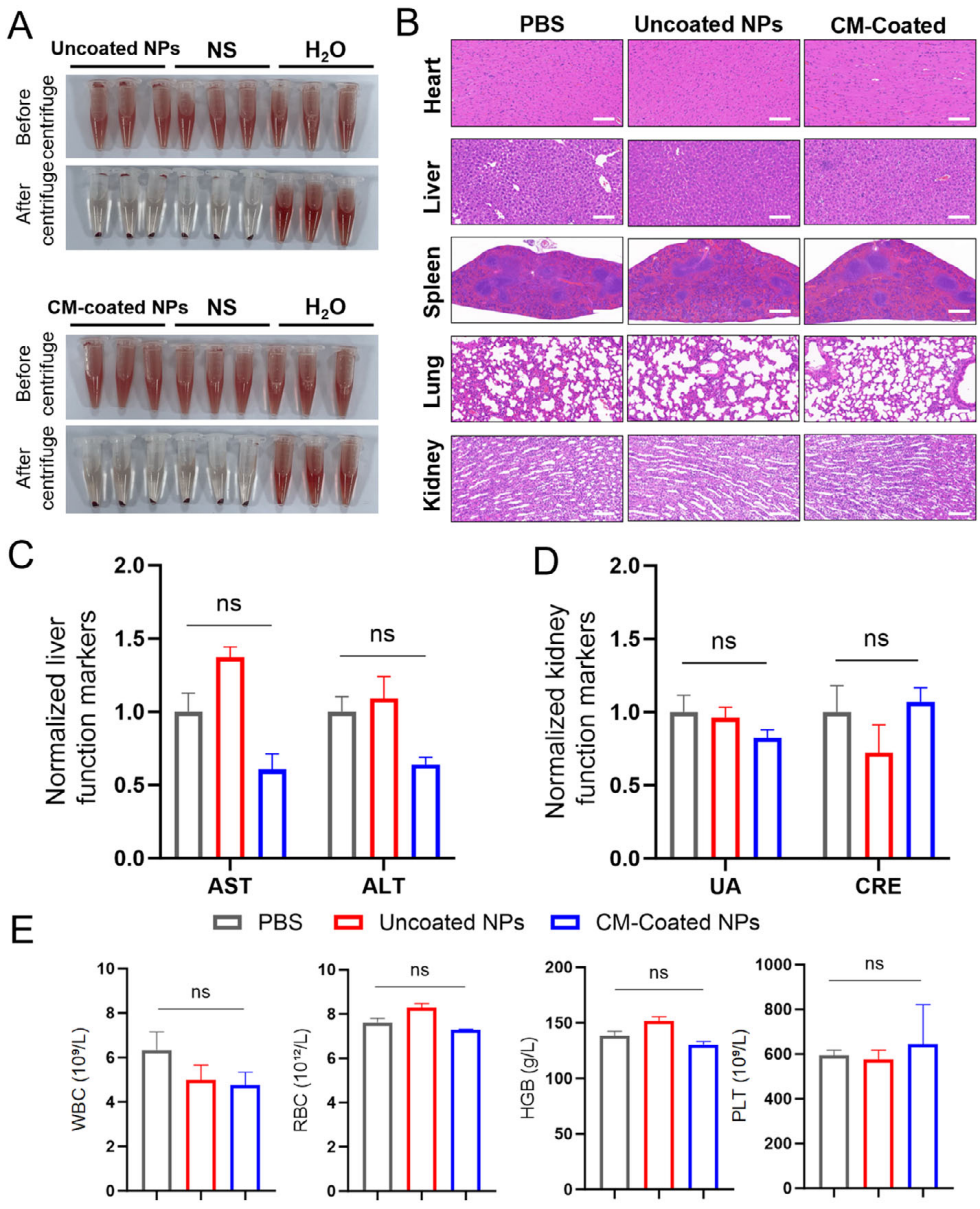
In vivo biosafety evaluation of PLL/TNA_AKT2_@468CM NPs. (A) Hemolysis assay of uncoated and CM‐Coated NPs. Ultrapure water and 1× PBS served as positive and negative controls, respectively. Neither formulation induced detectable hemolysis, confirming excellent hemocompatibility. (B) H&E staining of major organs (heart, liver, spleen, lungs, kidneys) harvested from all treatment groups, showing no detectable histological abnormalities or lesions, demonstrating the excellent biosafety profile of the biomimetic nanomedicine. Scale bars: 100 µm.Serum biochemistry analysis for (C) hepatic function markers (ALT and AST) and (D) renal function markers (UA and CRE). (E) Complete blood count parameters, including WBC, RBC, HGB, and PLT. Data are presented as mean ± SEM (n = 3). Statistical significance was assessed via one‐way ANOVA (ns: not significant).

To evaluate the hemocompatibility of the nanoparticles, we conducted a hemolysis assay by incubating both uncoated NPs and CM‐Coated NPs with red blood cells for 4 h in 37°C, utilizing ultrapure water and 1× PBS as positive and negative controls, respectively (Figure 10A and Figure S11). Neither formulation induced detectable hemolysis, confirming that the nanoparticles do not disrupt erythrocyte membrane integrity. To further assess the systemic safety profile and biocompatibility of the biomimetic nanomedicine, comprehensive histological and functional biochemical analyses were performed on tumor tissues and major organs harvested at the study endpoint. To evaluate the systemic impact of long‐term (30‐day) administration, histological examination of vital organs—including the heart, liver, spleen, lung, and kidney—was performed via H&E staining. Detailed analysis revealed no discernible pathological changes, such as inflammatory infiltrates, vacuolar degeneration, or structural abnormalities, in any of the treatment group (Figure 10B). The architectural integrity of these vital organs remained indistinguishable from that of the PBS control group, confirming the excellent biocompatibility and lack of acute or chronic organ toxicity associated with the membrane‐coated delivery system. This high level of safety is consistent with the stable body weight measurements (Figure 9E) and the absence of treatment‐related adverse behavioral effects throughout the study. Furthermore, functional biochemical assessments including serum biochemistry and complete blood count (CBC) analyses were conducted. As shown in Figure 10C,D, the levels of hepatic markers (ALT, AST) and renal function markers (UA, CRE) remained within normal physiological ranges for all treatment groups, with no significant differences observed compared to the PBS control group. Furthermore, hematological parameters including white blood cell (WBC), red blood cell (RBC) counts, hemoglobin (HGB), and platelet (PLT) showed no evidence of systemic inflammation, myelosuppression or anemia (Figure 10E).

Taken together, these in vivo results providing robust evidence that our biomimetic platform exhibits excellent hemocompatibility and no detectable systemic toxicity at the therapeutic doses. By combining the cell‐membrane clocking and TNA technology to target the AKT2 which is a key survival node and driver of metastasis in drug‐resistant TNBC [67], our platform achieved a **∼** 60% reduction in tumor volume growth compared to controls. Critically, our platform demonstrated a superior safety profile and broader generalizability compared to systemic chemotherapy. Unlike traditional regimens, which are typically associated with significant weight loss and dose‐limiting organ toxicity, our PLL/TNA_AKT2_@468CM NPs treated mice exhibited no loss in body weight or histological signs of systemic distress. This highlights a significantly wider therapeutic index and underscores the platform's potential to address clinical “unmet needs” where the standard of care fails to provide an effective, nono‐toxic solution for the general patient population.

The priority of selecting PLL/TNA_AKT2_@468CM NPs for in‐depth molecular analysis and in vivo studies was a strategic choice based on the following scientific and clinical considerations. First, the primary objective of this study is to develop a platform capable of overcoming the therapeutic barriers inherent in drug‐resistant TNBC. MDA‐MB‐468 cells represent the Basal‐like 1 (BL1) subtype, which is clinically associated with significant intrinsic resistance to standard‐of‐care chemotherapeutics and limited susceptibility to conventional nucleic acid transfection. MDA‐MB‐231 cells, while also a TNBC model, represent the Mesenchymal subtype and generally exhibit higher permeability to free oligonucleotides. By focusing on the 468 model, we chose the more rigorous “proof‐of‐concept” challenge. Demonstrating potent AKT2 silencing and tumor regression in this highly resistant model provides stronger evidence for the translational potential of our biomimetic platform. Second, the inclusion of both PLL/TNA_AKT2_@468CM NPs and PLL/TNA_AKT2_@231CM NPs in the initial characterization was intended to demonstrate the universality and modularity of our “plug‐and‐play” architecture. Once we confirmed that the homotypic targeting principle was operational and specific across both cell lines (as shown in the uptake and heterotypic cross‐pairing assays), we determined that a full duplication of all downstream molecular assays (Western blot, RT‐qPCR, etc.) in the MDA‐MB‐231 model would be redundant. Instead, we concentrated our resources on providing a multi‐layered functional analysis (viability, apoptosis, and signaling pathways) within the more clinically challenging MDA‐MB‐468 subtype. Furthermore, conducting parallel in vivo xenograft studies in multiple cell line models requires significant animal usage. Following the 3Rs principle (Replacement, Reduction, and Refinement), the MDA‐MB‐468 xenograft was selected as the most relevant model to validate the platform's ability to overcome drug resistance in a complex physiological environment. Since the MDA‐MB‐231 model does not present the same resistant barrier, its inclusion in the in vivo arm would not have provided proportionate scientific gain beyond the already established homotypic targeting data.

## 3 Conclusion

In summary, this study presents a new biomimetic nanomedicine platform, PLL/TNA_AKT2_@468CM NPs, that successfully integrates TNA‐mediated gene therapy with cell membrane technology to overcome the primary barriers in TNBC treatment. By employing a “top‐down” coating approach, we have characterized a unique fusion‐mediated entry mechanism that effectively bypassed the “endosomal bottleneck”, ensuring the direct cytosolic delivery and high bioavailability of chemically stable TNA polymers. This results in a breakthrough in biomimetic delivery that ensures the maximum integrity and potency of the genetic payload. Our findings demonstrate that this platform provides unprecedented intra‐disease discrimination, selectively targeting MDA‐MB‐468 cells over other TNBC subtypes and non‐malignant tissues. This high degree of homotypic fidelity translated into significant in vivo efficacy, achieving a 60% reduction in tumor volume in drug‐resistant xenograft models via potent AKT2 silencing. Importantly, the platform exhibited a superior safety profile with no systemic toxicity, representing a significant advancement over conventional chemotherapy. Ultimately, this work provides a strategic blueprint for the development of modular, “stealth” genetic therapeutics capable of addressing the molecular heterogeneity and clinical unmet needs of treatment‐refractory cancers.

## 4 Experimental Section

### 4.1 Materials

Tris(hydroxymethyl)aminomethane (Tris), ammonium persulfate, acrylamide/bisacrylamide 40% solution, skim milk powder, and Coomassie brilliant blue R250 were purchased from Sangon Biotech (Shanghai, China). Dulbecco's modified Eagle's medium (DMEM), TrypLE Express Enzyme, phosphate‐buffered saline (PBS), radioimmunoprecipitation assay (RIPA) lysis buffer, fetal bovine serum (FBS), horse serum, penicillin‐streptomycin, Stains‐All, Annexin V‐APC and propidium iodide (PI) were obtained from Thermo Fisher Scientific (USA). NanoVan (methylamine vanadate) was purchased from Nanoprobes (USA). Poly‐L‐lysine (PLL, MW 150 000–300 000 Da), BCA protein assay kit, phenylmethanesulfonyl fluoride (PMSF), Colored Pre‐stained Protein Molecular Weight Standard, and 1,1'‐dioctadecyl‐3,3,3',3'‐tetramethylindodicarbocyanine (DiD), 3,3'‐dioctadecyloxacarbocyanine perchlorate (DiO), 1,1'‐dioctadecyl‐3,3,3',3'‐tetramethylindocarbocyanine perchlorate (DiI) were acquired from Beyotime Biotechnology (Shanghai, China). Methyl‐β‐cyclodextrin (MβCD) were purchased from MedChemExpress (MCE, USA). Tetramethylethylenediamine (TEMED), dimethyl sulfoxide (DMSO), Tween‐20, amiloride hydrochloride, chlorpromazine hydrochloride, colchicine, sodium azide, heparin sodium, doxorubicin hydrochloride, hydrocortisone, cholera toxin, epidermal growth factor (EGF), insulin, LysoTracker, and MitoTracker were ordered from Sigma–Aldrich (USA). Primary and secondary antibodies against AKT2 were purchased from Cell Signaling Technology (USA). Polyvinylidene fluoride (PVDF) membranes (0.22 µm) were obtained from Merck Millipore (USA). Deionized (DI) water was purified using a Milli‐Q synthesis system (Millipore, USA). All chemical reagents were of analytical grade and used without further purification.

### 4.2 Instrumentation

The morphological and hydrodynamic properties of the nanoparticles were characterized by transmission electron microscopy (TEM; Philips Technai 12) and dynamic light scattering (DLS; Malvem Zetasizer Nano ZS), respectively. TNA_AKT2_ and FAM‐labeled TNA_AKT2_, primers of GAPDH and AKT2 were snthesized via standard automated solid‐phase synthesis on a MerMade MM6 DNA synthesizer (BioAutomation). UV–vis absorption spectra was recorded using a NanoDrop One C spectrophotometer (Thermo Scientific), and fluorescence measurements were performed on Fluormax‐4 Spectrofluorometer (HORIBA Jobin YvonTM). Confocal fluorescence scanning microscopy (CLSM) was conducted using a Leica TCS SP5 system equipped with 63× magnification. A Bio Tek Powerwave XS microplate reader was used for MTT cell viability assays and protein quantification (BCA assay). Cellular uptake and internalization were quantified using a CytoFLEX (B5‐R3‐V5) flow cytometer (Beckman Coulter). cDNA synthesis and quantitative real‐time PCR (qRT‐PCR) were performed using a SimpliAmp Thermal Cycler (Applied Biosystems) and a CFX‐96 Real Time PCR Detection System (Bio‐Rad), respectively. Western blot protein bands were visualized using a LAS‐4000 luminescent image analyzer (Fujifilm), and general gel imaging was performed on a ChemiDoc Touch Imaging System (Bio‐Rad, USA). For histological analysis, tissues were processed using an Excelsior AS tissue processor (Thermo Scientific), a YB‐6LF embedding center (Xiaogan Yaguang), and an HM 340E microtome (Thermo Scientific). Histological sections were stained with a Gemini AS automated slide Stainer (Thermo Scientific) and digitized using a 3D Pannoramic MIDI slide scanner. Serum biochemistry and complete blood count (CBC) analyses were conducted using an automated biochemical analyzer and an automated hematology analyzer, respectively.

### 4.3 Cell Culture

The TNBC cell lines MDA‐MB‐468 (Basal‐like 1 TNBC) and MDA‐MB‐231 (Mesenchymal stem‐like TNBC), (the doxorubicin‐resistant breast cancer line MCF‐7/ADR (ER/PR‐positive), and the non‐tumorigenic human mammary epithelial cell line MCF‐10A were purchased from the American Type Culture Collection (ATCC). MDA‐MB‐468 and MDA‐MB‐231 cells were maintained in Dulbecco's Modified Eagle Medium (DMEM) supplemented with 10% FBS, and 1% penicillin‐streptomycin (100 U/mL penicillin and 100 µg/mL streptomycin). MCF‐7/ADR cells were cultured in RPMI‐1640 medium supplemented with 10% FBS, 1% penicillin‐streptomycin. The MCF‐10A cells were grown in DMEM/F12 medium supplemented with 5% horse serum, 20 ng/mL epidermal growth factor (EGF), 10 µg/mL insulin, 0.5 µg/mL hydrocortisone, 100 ng/mL cholera toxin, and 1% penicillin‐streptomycin. All cell lines were cultured in humidified incubator at 37°C with 5% CO_2_.

### 4.4 Synthesis and Characterization of TNA

TNA oligonucleotides were synthesized via an automated solid‐phase phosphoramidite chemistry following established protocols [41]. The identity and molecular weight of the synthesized TNA sequences were confirmed by matrix‐assisted laser desorption/ionization time‐of‐flight (MALDI‐TOF) mass spectrometry. TNA concentrations were determined by UV–vis spectrophotometry at 260 nm using a Nanodrop OneC spectrophotometer.

### 4.5 Preparation of PLL/TNA_AKT2_ Nanoparticles (PLL/TNA_AKT2_ NPs)

PLL/TNA_AKT2_ NPs were prepared by mixing equal volumes of PLL and TNA_AKT2_ aqueous solutions at various nitrogen‐to‐phosphorus (N/P) ratios ranging from 0.2:1 to 10:1. The resulting mixtures were vortexed thoroughly for 1 min and incubated at 4°C for 2 h to facilitate electrostatic self‐assembly. TNA condensation efficiency was subsequently evaluated by agarose gel electrophoresis (0.5%–1% w/v) under native conditions (30 mA, 25 min). The gels were visualized and analyzed using a Bio‐Rad imaging system.

### 4.6 Extraction of Cancer Cell Membranes

MDA‐MB‐468 and MDA‐MB‐231 cells were washed with pre‐chilled PBS and harvested via gently mechanical detachment using a cell scraper. The resulting cell suspensions were centrifuged at 1000 rpm for 5 min at 4°C. The cell pellet was then resuspended in a hypotonic lysis buffer (1 mM NaHCO_3_, 0.2 mm EDTA, 75 mM sucrose) supplemented with an EDTA‐free protease inhibitor cocktail and incubated for 20 min at 4°C. Following the addition of phenylmethylsulfonyl fluoride (PMSF), the cells were mechanically disrupted using a Dounce tissue grinder. The homogenate was centrifuged at 3200 rpm for 5 min at 4°C to remove nuclei, unbroken cells and debris. The resulting supernatant was subjected to a differential centrifugation protocol: (1) 15 000 × g for 20 min at 4°C to remove mitochondria and other large organelles, followed by (2) 100 000 × g for 70 min at 4°C to pellet the purified membrane fraction. The isolated membranes were resuspended in PBS and stored at −20°C for further use.

### 4.7 Preparation of PLL/TNA_AKT2_@468CM NPs and PLL/TNA_AKT2_@231CM NPs

The biomimetic nanoparticles, PLL/TNA_AKT2_@468CM NPs and PLL/TNA _AKT2_@231CM NPs were prepared by mixing 0.5 mL of purified cell membrane solution (40 µg/mL) with 0.5 mL of PLL/TNA _AKT2_ NP solution (50 µM TNA _AKT2_ concentration). The mixture was sequentially extruded 11 times through polycarbonate membranes with pore sizes of 800, 400, and 200 nm using a mini‐extruder. FAM‐labeled biomimetic NPs were synthesized via the same protocol using FAM‐labeled TNA_AKT2_. The final process yield was determined by a fluorescence quenching‐recovery assay. To quantify the encapsulated cargo, the membrane shell disrupted with 50% DMSO, and the TNA_AKT2_ was subsequently released from the PLL carrier using heparin sodium (5 mg/mL), a concentration validated to achieve quantitative displacement. The restored FAM fluorescence was measured and quantified against a standard calibration curve (R^2^ > 0.99).

### 4.8 Colocalization Assay

Purified MDA‐MB‐468 cell and MDA‐MB‐231 cell membranes were incubated with DiD (5 µm) at 37°C for 30 min. Excess dye was removed via ultrafiltration at 4000 rpm using a centrifugal filter prior to nanoparticle fabrication. PLL/TNA_AKT2_@468CM NPs were then prepared using the DiD‐labeled membranes and FAM‐labeled TNA_AKT2_. The resulting dual‐labeled nanoparticles were deposited onto a glass coverslip and the colocalization of the DiD (membrane shell) and FAM (TNA cargo) fluorescence signals was visualized by confocal laser scanning microscopy (CLSM). Pearson's correlation coefficient (PCC) was calculated from the acquired images using ImageJ software to quantify the degree of overlap.

### 4.9 SDS‐PAGE Protein Analysis

Pure cell membrane, cell membrane vesicles, and PLL/TNA_AKT2_@CM NPs were lysed in RIPA buffer, and protein concentration was determined via BCA assay. Equal amount of protein (30 µg) was mixed with loading buffer, denatured, and loaded onto 10% sodium dodecyl sulfate‐polyacrylamide gel electrophoresis (SDS‐PAGE) gels. After electrophoresis, the proteins were visualized by staining with Coomassie Brilliant Blue.

### 4.10 TEM Characterization

Sample solutions (4 µL) were deposited onto carbon‐coated copper grids and allowed to air‐dried. Negative staining was performed by applying 5 µL of NanoVan staining solution for 1 min, after which the excess liquid was carefully blotted with filter paper. The grids were then rinsed with autoclaved water to remove residual salt and contaminants. The sample were imaged using a Tecnai 12 TEM (Philips) operating at an acceleration voltage of 120 kV.

### 4.11 Stability of PLL/TNA_AKT2_ NPs and PLL/TNA_AKT2_@468CM NP

PLL/TNA_AKT2_ NPs and PLL/TNA_AKT2_@468CM NPs were incubated at 37°C in either 1 X PBS or DMEM supplemented with 10% FBS. The hydrodynamic diameter and polydispersity index (PDI) were monitored via DLS at predetermined time points over a period of 12 days.

### 4.12 Denaturing PAGE Analysis of TNA Integrity

The FAM‐labeled TNA was extracted from PLL/TNA_AKT2_@468CM NPs as described in Experimental Section 2.7. The released FAM‐TNA (0.05 OD per 15 µL) was analyzed via 8% denaturing polyacrylamide gel electrophoresis (PAGE) containing 7 m urea, operated at 200 V for 20 min. Two control groups were included: (1) native FAM‐labeled TNA_AKT2_ (untreated control) and FAM‐TNA_AKT2_ incubated with the release agents (50% DMSO and 5 mg/mL heparin). The gel was visualized using Stains‐All dye and imaged with the ChemiDoc Touch Imaging System (Bio‐Rad).

### 4.13 Homotypic and Heterotypic Targeting Analysis

For FCM analysis, MDA‐MB‐468 and MDA‐MB‐231 cells were seeded in 24‐well plates at a density of 8 × 10^4^ cells/well and allowed to adhere overnight. Following a 24 h incubation with various formulations, the cells were harvested, washed with PBS, and quantified using a CytoFLEX flow cytometer.

For CLSM imaging, MDA‐MB‐468 and MDA‐MB‐231 cells were seeded in confocal dishes (3 × 10^4^ cells/dish) and incubated overnight. After treatment with the respective formulations for 24 h, the cells were incubated with LysoTracker Red according to the manufacture's instruction. The cells were then washed and imaged using a Leica TCS SP5 confocal microscope.

### 4.14 Cell Uptake Mechanism Study of PLL/TNA_AKT2_@468CM NPs

MDA‐MB‐468 cells were seeded in 24‐well plates at a density of 8 × 10^4^ cells/well and allowed to adhere overnight. The cells were then pre‐treated with various pharmacological inhibitors in serum‐free DMEM prior to nanoparticle exposure: chlorpromazine (2.5 mm, 1 h); colchicine (10 µm, 1 h); NaN_3_ (10 mm, 1 h) amiloride (5 µm, 1 h); low‐dose MβCD (0.5 mm, 1 h); high‐dose MβCD (10 mm, 30 min). Additionally, a group was pre‐incubated at 4°C (1 h). Following pre‐treatment, the cells were incubated with FAM‐labeled PLL/TNA_AKT2_@468CM NPs (200 nm TNA concentration) for 24 h and subsequently analyzed via flow cytometry.

### 4.15 Selectivity Uptake of PLL/TNA_AKT2_@468CM NPs

MCF‐7/ADR cells and MCF‐10A cells were seeded in 24‐well plates at a density of 6 × 10^4^ cells/well and incubated overnight. The cells were then treated with FAM‐labeled PLL/TNA_AKT2_@468CM NPs (200 nm TNA concentration) for a duration of 6 h. A shorter incubation period was specifically selected to capture the initial homotypic recognition phase and minimize the influence of non‐specific binding or saturation effects. Following treatment, the cells were harvested, washed with PBS, and analyzed via flow cytometry (FCM) as previously described.

### 4.16 FRET‐Based Lipid Mixing Assay

The membrane shell of PLL/TNA_AKT2_@468CM NPs was labeled with DiO by incubating purified MDA‐MB‐468 cell membranes with 5 µm DiO for 30 min at 37°C prior to extrusion process. MDA‐MB‐468 cells were seeded in confocal dishes (3 × 10^4^ cells/dish) and cultured overnight. The plasma membrane of live cells was stained with DiI at 5 µm for 15 min at 37°C, followed by three washes with PBS.

For the inhibition group, cells were pre‐treated with 10 mm MβCD in serum‐free DMEM for 30 min at 37°C prior to DiI staining. DiO‐labeled PLL/TNA_AKT2_@468CM NPs were added to the DiI‐stained cells at a final TNA concentration of 200 nm. Time‐lapse imaging was initiated 55 min after nanoparticle addition. FRET signals (sensitized DiI emission upon 488 nm excitation) were recorded for 5 min using a Leica TCS SP5 confocal microscope equipped with a 63× magnification objective.

### 4.17 Quantitative Reverse Transcription Polymerase Chain Reaction (qRT‐PCR) Analysis

MDA‐MB‐468 cells were seeded in 6‐well plates (5 × 10^5^ cells/well) and incubated overnight. Cells were treated with PBS, TNA _AKT2_, Lipofectamine‐transfected TNA _AKT2_, PLL/TNA _AKT2_ NPs, PLL/TNA _AKT2_@468CM NPs, or PLL/TNA_SCR_@468CM NPs (200 nM TNA concentration) for 48 h. Total RNA was extracted using commercial RNA extraction kits and reverse transcribed into cDNA. RT‐qPCR was performed using SYBR Green master mix on a Bio‐Rad CFX‐96 system. Gene expression was normalized to GAPDH and calculated using the 2^−^ΔΔCt method. Primer sequences:

AKT2 Forward: 5’‐ACCACAGTCATCGAGAGGACC‐3’;

AKT2 Reverse: 5’‐GGAGCCACACTTGTAGTCCA‐3’;

GAPDH Forward: 5’‐GGTGAAGGTCGGTGTGAACG‐3’;

GAPDH Reverse: 5’‐CTCGCTCCTGGAAGATGGTG‐3’.

### 4.18 Western Blot (WB) Analysis

MDA‐MB‐468 cells were seeded in 6‐well plates (5 × 10^5^ cells/well) and treated with the indicated formulations (200 nm TNA concentration) for 72 h. Cells were lysed in RIPA buffer containing protease inhibitors, and protein concentration was determined by BCA assay. Equal protein amounts (30 µg) were separated by 10% SDS‐PAGE and transferred to PVDF membranes. Membranes were blocked with 5% skim milk in TBST, incubated overnight with primary antibodies against AKT2 and β‐actin at 4°C, followed by HRP‐conjugated secondary antibodies for 1 h at room temperature. Protein bands were visualized by chemiluminescence and quantified using ImageJ software.

### 4.19 MTT Assays

MDA‐MB‐468 cells were seeded in 96‐well plates (1 × 10^4^ cells/well) and incubated overnight. Cells were treated with various formulations (TNA concentration: 200 nm) for 24, 48, or 72 h. MTT solution (20 µL, 5 mg/mL) was added to each well and incubated for 4 h. Formazan crystals were dissolved in 100 µL DMSO, and absorbance was measured at 570 nm using a microplate reader. Cell viability was calculated as percentage relative to untreated controls.

### 4.20 Apoptosis Assay

MDA‐MB‐468 cells were seeded in 6‐well plates (3 × 10^5^ cells/well) and treated with free TNA_AKT2_, PLL/TNA_AKT2_ NPs, or PLL/TNA _AKT2_@468CM NPs (TNA concentration: 200 nm) for 48 h. Cells were harvested, washed with cold PBS, and stained with Annexin V‐APC and propidium iodide (PI) in binding buffer for 15 min at room temperature in the dark. Apoptotic cells were quantified by FCM. Data were analyzed using FlowJo software.

### 4.21 Hemolysis Assay

Fresh murine blood was collected from BALB/c nude mice into heparinized tubes. To isolate the erythrocytes, the anticoagulated whole blood was centrifuged at 1000 rpm for 10 min. The resulting red blood cell pellet (0.2 mL) was transferred to a 1.5 mL centrifuge tube, washed with 1 mL of physiological saline, and gently mixed by inversion. Following centrifugation at 1000 rpm for 10 min, the supernatant was removed, and the washing step was repeated. The final RBC pellet was diluted to 5% (v/v) suspension in physiological saline. For the assay, 500 µL of 5% RBC suspension was mixed with 500 µL of PLL/TNA_AKT2_ NPs or PLL/TNA_AKT2_@468CM NPs in PBS (final TNA concentration: 200 nm). Ultrapure water and 1× PBS served as the positive and negative controls, respectively. The samples were incubated at 37°C for 4 h with gentle shaking, followed by centrifugation at 1,000 × g for 10 min. The absorbance of the supernatant (released hemoglobin) was measured at 545 nm using a microplate reader. The hemolysis percentage was calculated as:

$$\mathrm{Hemolysis}\left(\%\right)=\frac{\left(A_{\mathrm{sample}}-A_{\mathrm{PBS}}\right)}{\left(A_{\mathrm{water}}-A_{\mathrm{PBS}}\right)}\times 100\%$$

where A_sample_, A_water_ and A_PBS_ are the absorbance values of the experimental sample, the negative control, and the positive control, respectively.

### 4.22 Animals

All animal experiments were approved by the Animal Ethics Committee of City University of Hong Kong and conducted in accordance with institutional guidelines. Female BALB/c nude mice (6–8 weeks old) were purchased from the Laboratory Animal Research Unit (LARU) at City University of Hong Kong and housed under specific pathogen‐free conditions.

For tumor xenograft establishment, MDA‐MB‐468 cells (5 × 10^6^ cells in 100 µL PBS mixed 1:1 with Matrigel) were subcutaneously injected into the mammary fat pad of mice. When tumors reached approximately 100 mm^3^, mice were randomly assigned to three groups (n = 4 per group): (1) PBS control, (2) PLL/TNA_AKT2_ NPs (Uncoated NPs), and (3) PLL/TNA _AKT2_@468CM NPs (CM‐Coated NPs) at a TNA dosage of 1 mg/kg. Treatments were administered via tail vein injection every 2 days for a total of 15 injections over 30 days. Tumor volume was calculated as (length × width^2^)/2. Body weight was monitored throughout the study.

### 4.23 In Vivo Biodistribution Imaging

MDA‐MB‐468 tumor‐bearing mice were intravenously injected with PLL/Cy5.5‐TNA_AKT2_ NPs (Uncoated NPs) or PLL/Cy5.5‐TNA_AKT2_@468CM NPs (CM‐Coated NPs). In vivo fluorescence imaging was performed at 1, 4, 8, and 24 h post‐injection using an IVIS Spectrum imaging system. At 4 and 24 h post‐injection, mice were euthanized, and major organs (heart, liver, spleen, lungs, kidneys) and tumors were harvested for ex vivo imaging. Fluorescence signals were quantified using AniView software.

### 4.24 Immunohistochemistry (IHC)

Tumor tissues were fixed in 4% paraformaldehyde, embedded in paraffin, and sectioned (4–7 µm thickness). Sections were deparaffinized, rehydrated, and subjected to antigen retrieval in citrate buffer (pH 6.0). Endogenous peroxidase activity was blocked with 3% H_2_O_2_. Sections were incubated with primary antibodies against AKT2, p21, and cleaved Caspase‐3 overnight at 4°C, followed by HRP‐conjugated secondary antibodies. Immunoreactivity was visualized using DAB substrate, and sections were counterstained with hematoxylin. Images were acquired using a light microscope.

### 4.25 Histological Analysis

Major organs (heart, liver, spleen, lungs, kidneys) and tumors were harvested, fixed in 4% paraformaldehyde, embedded in paraffin, and sectioned (5 µm thickness). Sections were stained with hematoxylin and eosin (H&E) for histopathological evaluation. TUNEL staining was performed using an in situ apoptosis detection kit according to the manufacturer's protocol to assess DNA fragmentation in tumor tissues.

### 4.26 Serum Biochemistry and Hematological Analysis

At the study's endpoint, whole blood was collected from the mice via cardiac puncture and partitioned into serum separator tubes and EDTA‐coated tubes for biochemical and hematological analyses, respectively. To obtain serum, the samples was allowed to clot and then centrifuged at 3000 × g for 15 min at 4°C. Liver function markers, including alanine aminotransferase (ALT) and aspartate aminotransferase (AST), and kidney function markers, including urea (UA) and creatinine (CRE), were measured using an automated biochemical analyzer. Comprehensive hematological parameters, including white blood cell (WBC) count, red blood cell (RBC) count, hemoglobin (HGB) concentration, and platelet (PLT) count, were determined using an automated hematology analyzer.

### 4.27 Statistical Analysis

All in vitro data are presented as mean ± standard deviation (SD) from at least three independent experiments. In vivo tumor growth data are presented as mean ± standard error of the mean (SEM). To ensure data integrity, tumor volume measurements were screened for statistical outliers using Grubbs' test (two‐sided, α = 0.05); no statistically significant outliers were identified in any experimental group. Statistical analyses were performed using GraphPad Prism and Origin software. Comparisons between two groups were evaluated using a two‐tailed Student's *t*‐test, while differences among multiple group were analyzed via one‐way analysis of variance (ANOVA) followed by Tukey's honestly significant difference (HSD) post‐hoc test for multiple comparisons. Statistical significance was defined as ^*^ *p* < 0.05, ^**^ *p* < 0.01, ^***^ *p* < 0.001.

## Author Contributions

**Wei Zheng**: conceptualization, methodology, investigation, validation, formal analysis, data curation. **Tristan Juin Han Chang**: methodology, conceptualization. **Pierre Karam**: investigation, validation, formal analysis. **Xinchao Li**: formal analysis, investigation. **Zhongqi Zhou**: formal analysis, investigation. **Kenward Vong**: conceptualization, formal analysis, funding acquisition. **Chung Tin**: investigation, validation, formal analysis. **Pik Kwan Lo**: conceptualization, investigation, funding acquisition, writing – original draft, visualization, validation, methodology, project administration, formal analysis, supervision.

## Conflicts of Interest

The technological innovations and biomimetic delivery strategies described in this manuscript have been filed for a US Provisional Patent (Title: *A Biomimetic Drug Delivery System by TNA Technology for Triple Negative Breast Cancer Therapy*; Priority No. 63/975,357; Filed: February 4, 2026).

## Supporting information




**Supporting File 1**: advs76100‐sup‐0001‐suppMat.docx.

**Supporting File 2**: advs76100‐sup‐0002‐VideoS1.mp4.

**Supporting File 3**: advs76100‐sup‐0003‐VideoS2.mp4.

**Supporting File 4**: advs76100‐sup‐0004‐VideoS3.mp4.

**Supporting File 5**: advs76100‐sup‐0005‐VideoS4.mp4.

## Data Availability

The data that support the findings of this study are available from the corresponding author upon reasonable request.
