# Supplementary material for: Membrane Fusion‐Mediated Cytosolic Delivery of Threose Nucleic Acids via Homotypic Nanoparticles Overcomes Drug Resistance in Triple‐Negative Breast Cancer
Source: Adv Sci (Weinh). 2026 Jun 12:e76100. Online ahead of print. doi: 10.1002/advs.76100 (PMC13337009; doi:10.1002/advs.76100)
Supplement: Supplementary file 1 — Supporting File 1: advs76100‐sup‐0001‐suppMat.docx. [file ADVS-9999-e76100-s005.docx]

Supporting information

**Membrane Fusion-Mediated Cytosolic Delivery of Threose Nucleic Acids via Homotypic Nanoparticles Overcomes Drug Resistance in Triple-Negative Breast Cancer**

*Wei Zheng, ^a^ Tristan Juin Han Chang, ^a^ Xinchao Li, ^a^ Zhongqi Zhou, ^a^ Chung Tin,^b^ Kenward Vong, ^c^* Pierre Karam, *^d^ and Pik Kwan Lo ^a,e,f^ **

^a^ Department of Chemistry and State Key Laboratory of Marine Environmental Health, ^b^ Department of Biomedical Engineering, City University of Hong Kong, Tat Chee Avenue, Kowloon Tong, Hong Kong SAR, China ^c^ Department of Chemistry, Hong Kong University of Science and Technology, Clear Water Bay, Kowloon, Hong Kong, China ^d^ Department of Chemistry, American University of Beirut, Beirut 1107 2020, Lebanon ^e^ City University of Hong Kong Chengdu Research Institute, Chengdu, China ^f^ Key Laboratory of Biotech and Health Care, Shenzhen Research Institute of City University of Hong Kong, 518057, Shenzhen, China

Email: [peggylo@cityu.edu.hk](mailto:peggylo@cityu.edu.hk)

**Table S1.** Sequences of TNA oligonucleotides used in this study.

| TNA_AKT2_ | 3’-GTCTTGATGTATTCACCACGC-2’ |
| --- | --- |
| TNA_SCR_ | 3’-TAGGATCTACACAGAGATGAG-2’ |


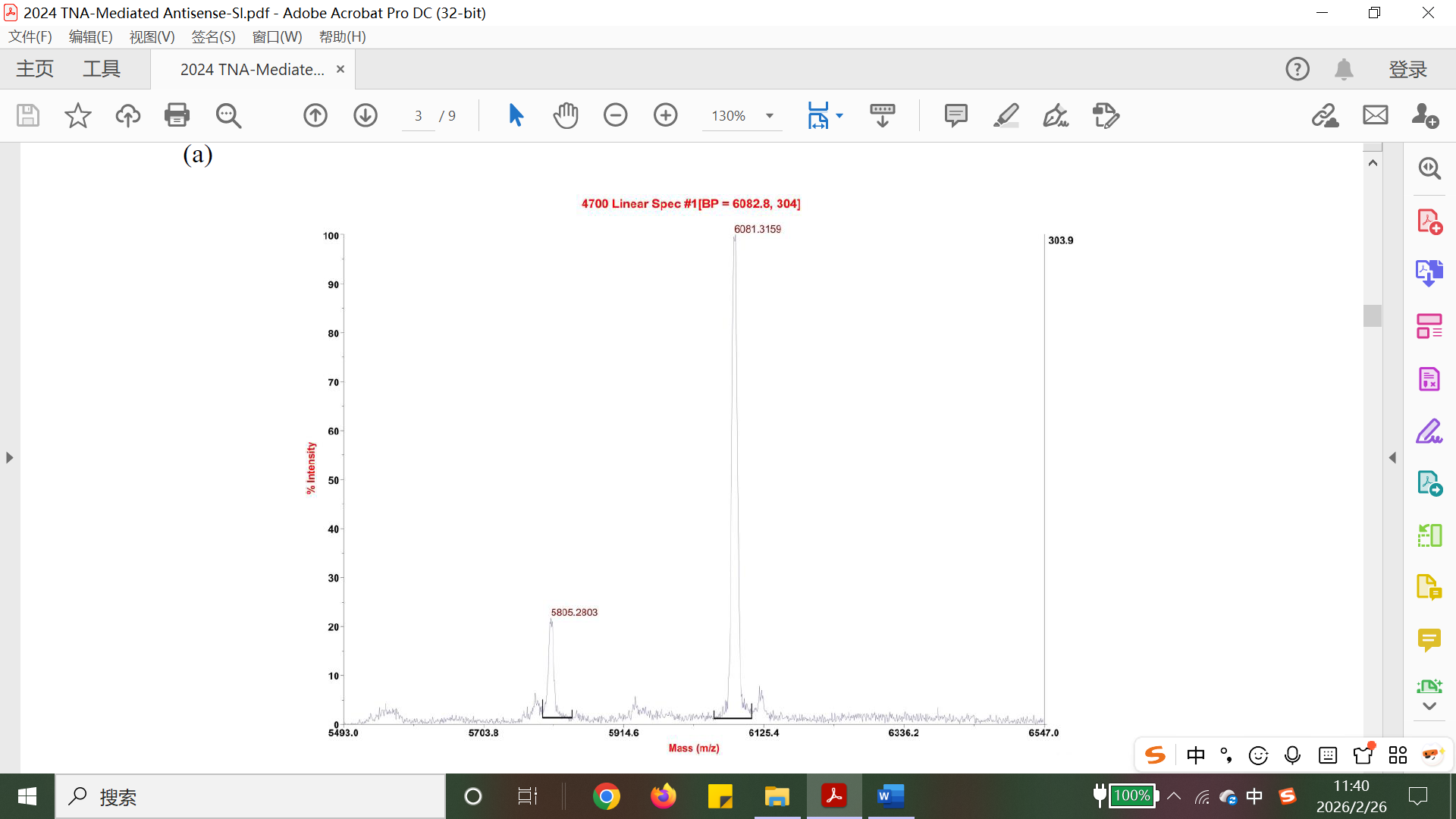


**Figure S1. MALDI-TOF mass spectrometry characterization of synthesized TN_AKT2_.** The observed molecular weight (m/z found: 6081.32) is consistent with the calculated value (m/z calc: 6136.44), confirming successful synthesis of the TNA_AKT2_ antisense oligonucleotide.


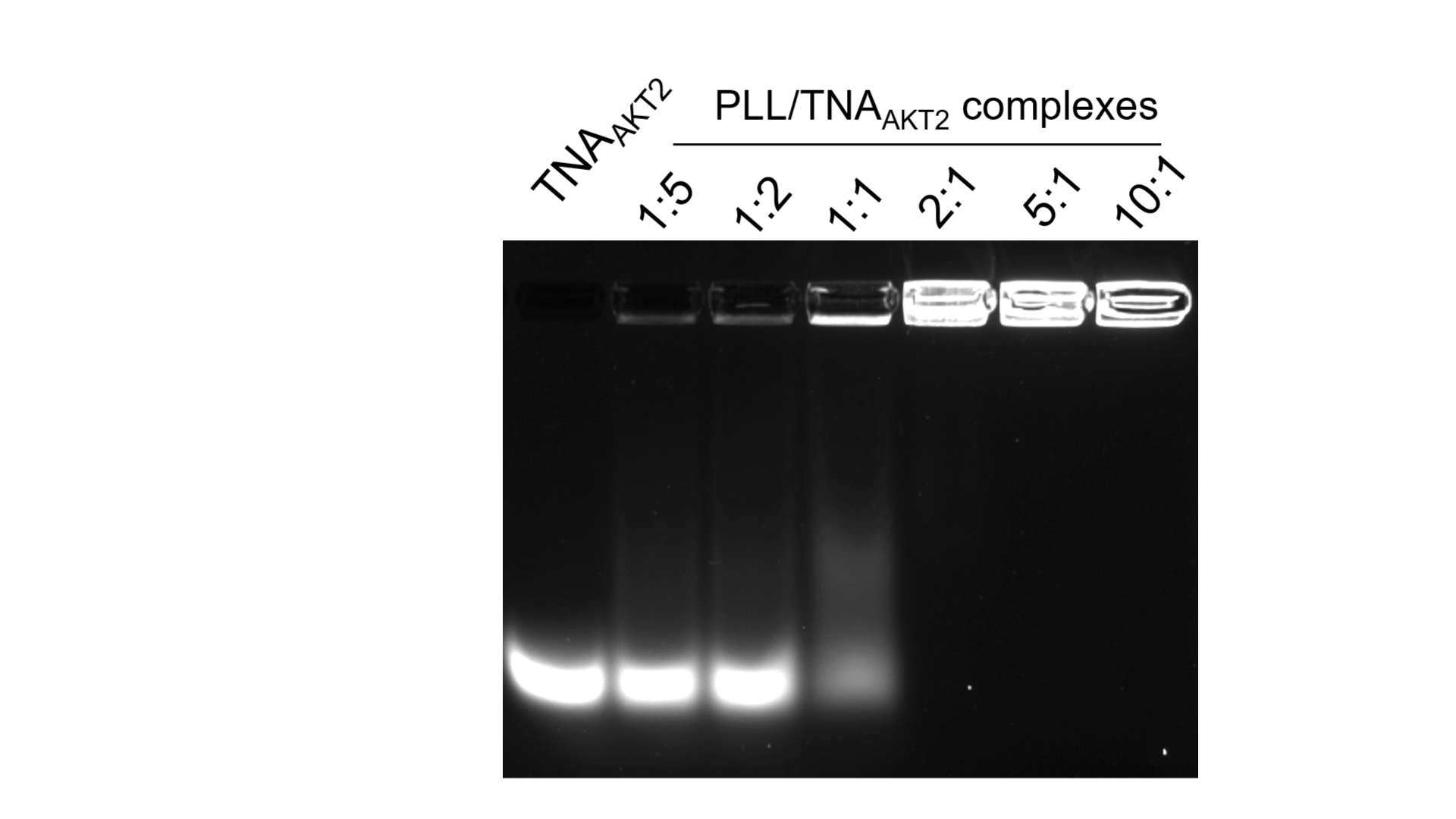


**Figure S2. Agarose gel electrophoresis analysis of PLL/TNA_AKT2_ complex formation.** 1% agarose gel retardation assay of PLL/TNA_AKT2_ complexes at various N/P ratios under native conditions. Complete TNA condensation is achieved at N/P ratios ≥ 2:1.


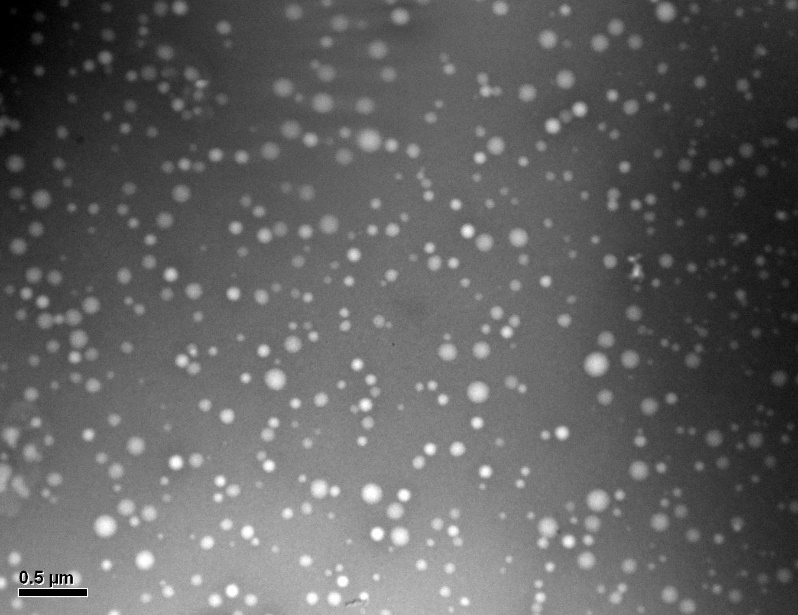


**Figure S3. Representative TEM image of PLL/TNA_AKT2_ nanoparticles at low magnification.** Negatively stained PLL/TNA_AKT2_ NPs show uniform spherical morphology with no apparent aggregation. Scale bar: 500 nm.


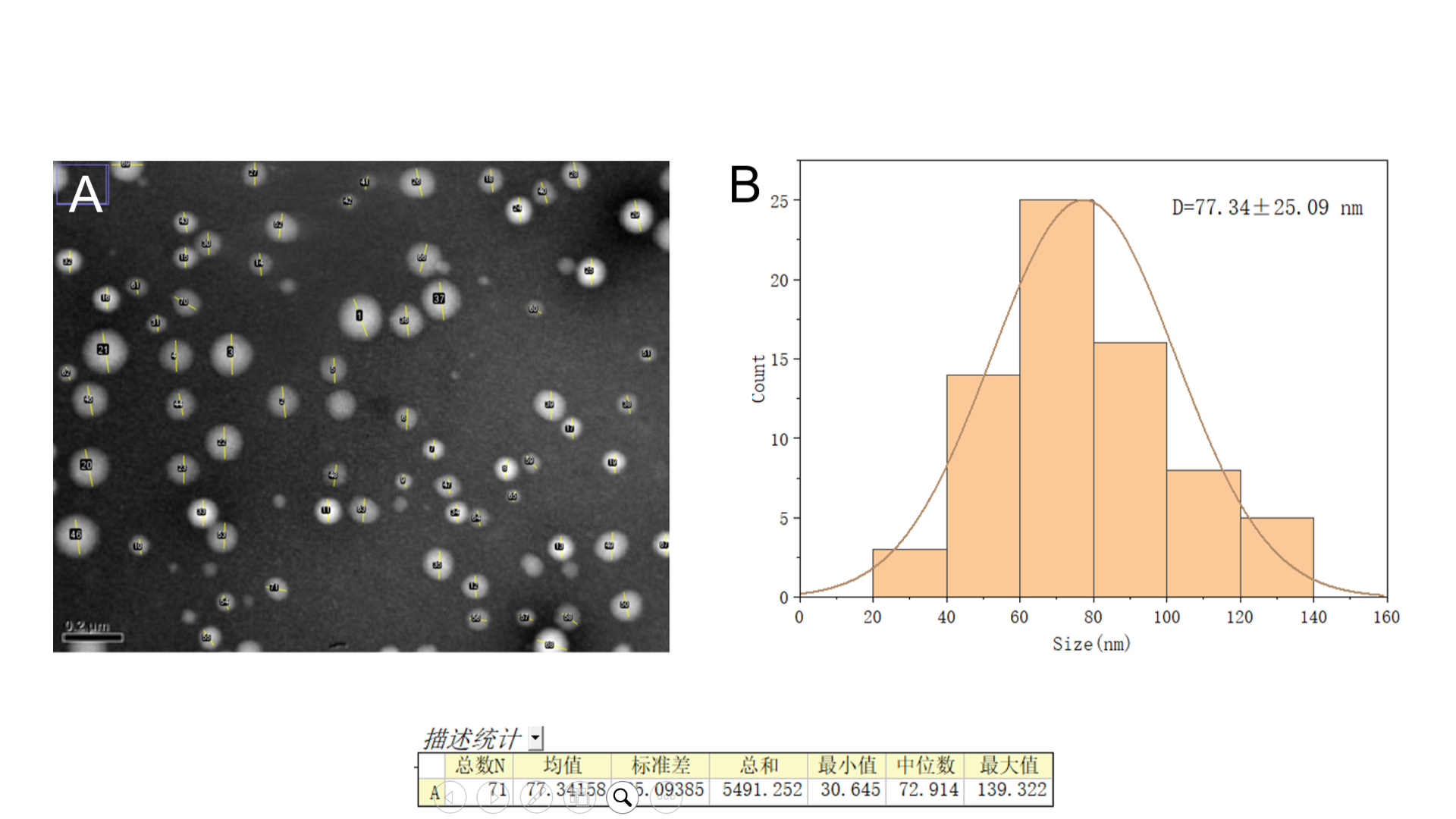


**Figure S4. Size distribution analysis of PLL/TNA_AKT2_ NPs from TEM images.** Statistical analysis of nanoparticle diameters (n > 100) reveals an average core size of 77.34 ± 25.09 nm, confirming uniform nanoparticle formation.


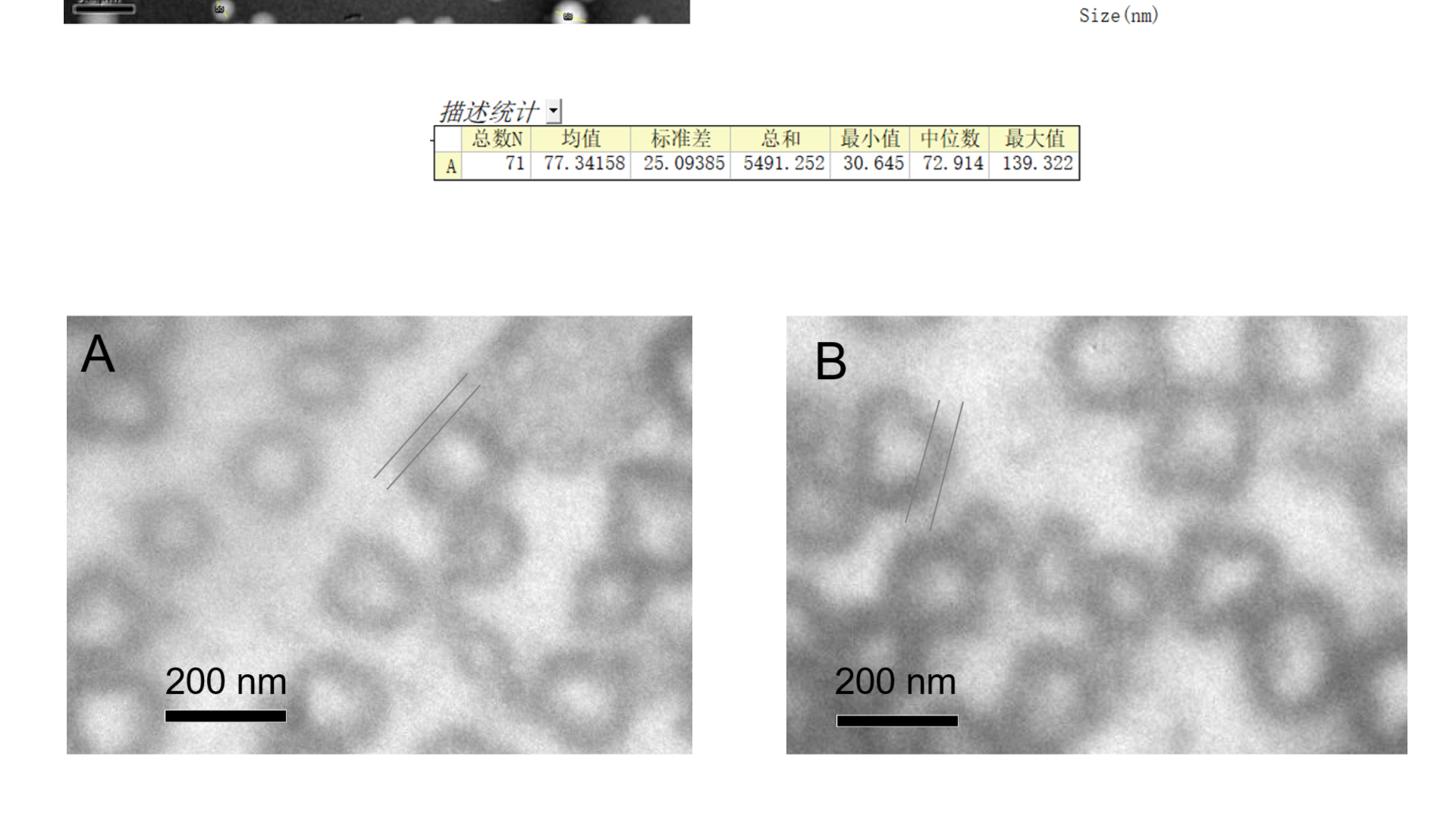


**Figure S5. TEM characterization of purified cancer cell membrane vesicles.** (A) Membrane vesicles derived from MDA-MB-468 cells. (B) Membrane vesicles derived from MDA-MB-231 cells. Both preparations show typical vesicular morphology with bilayer structures. Scale bars: 200 nm.


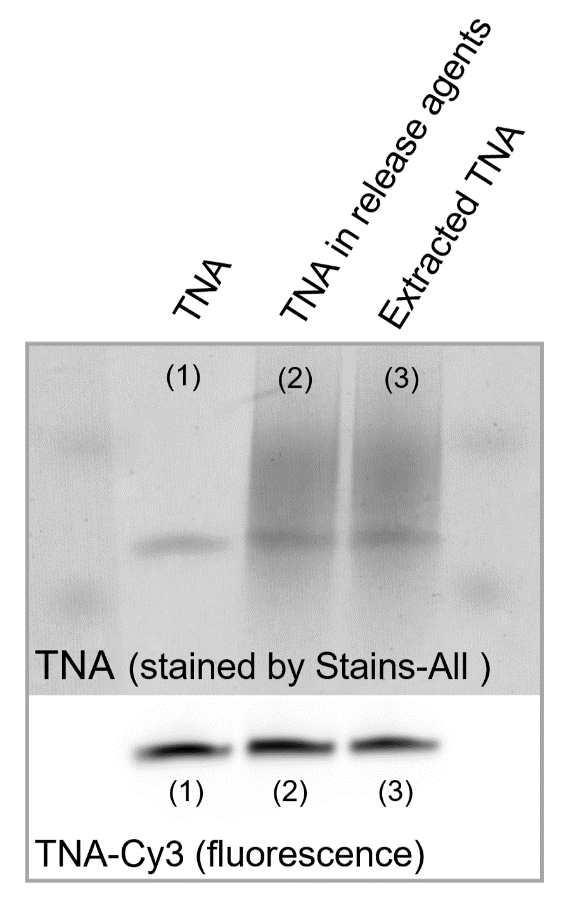


**Figure S6**. 8% Denaturing PAGE analysis of the structural integrity of Cy3-labeled TNA_AKT2_ during the nanoparticle fabrication process. Lane 1: Native TNA_AKT2_ (untreated control); Lane 2: TNA_AKT2_ incubated with heparin and DMSO (release agents); Lane 3: TNA_AKT2_ extracted from PLL/TNA_AKT2_@468CM NPs post-fabrication.


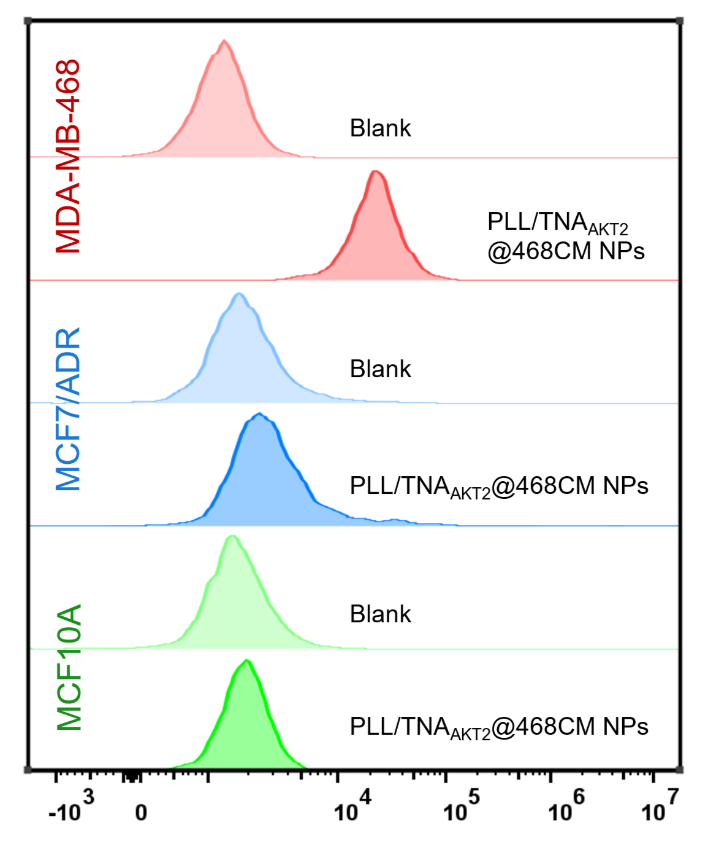


**Figure S7**. Flow cytometry analysis of PLL/TNA_AKT2_@468CM NPs uptake in MDA-MB-468, MCF-7/ADR and MCF-10A cells after 6 h treatment.


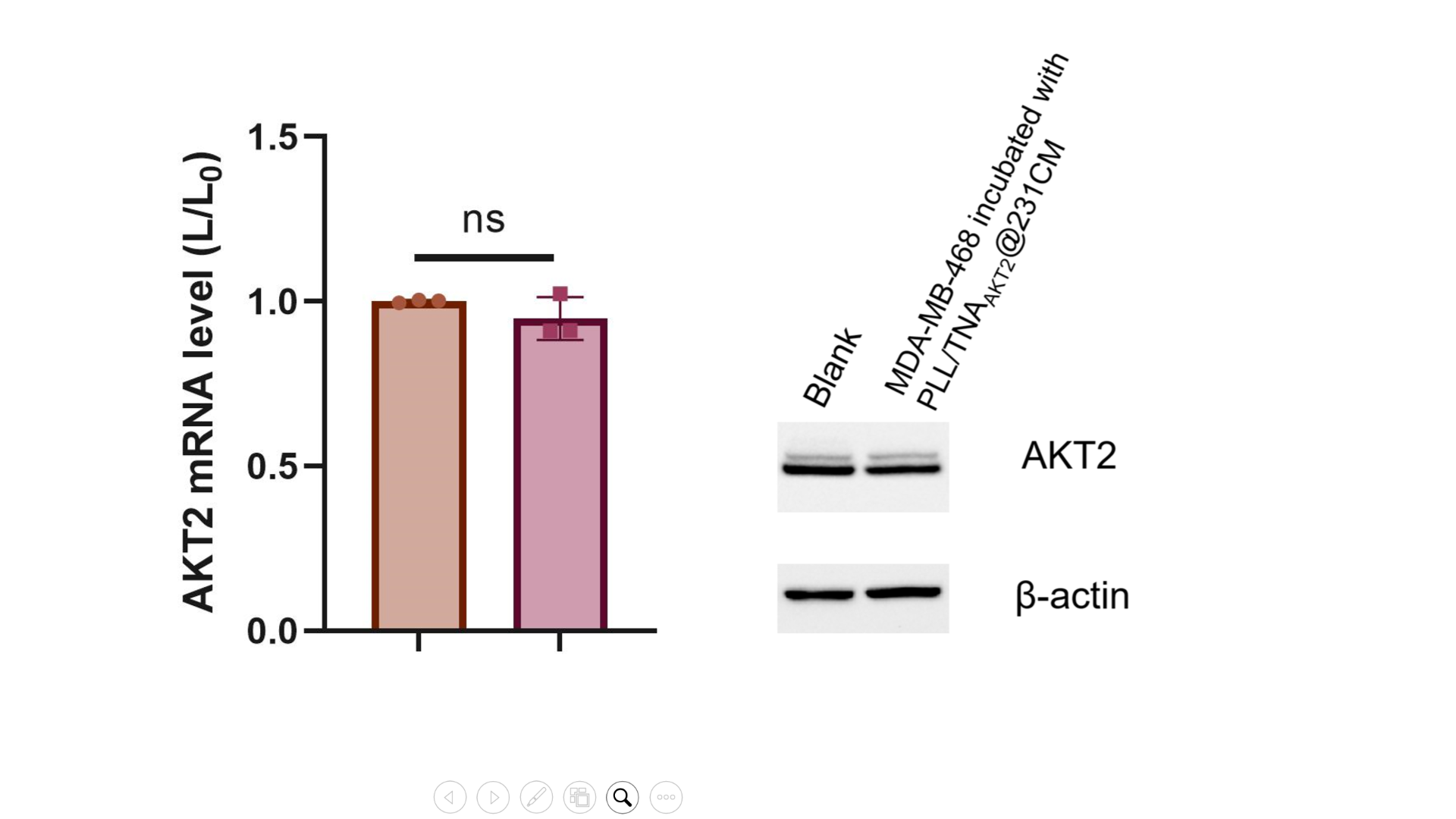


**Figure S8. Heterotypic targeting fails to induce AKT2 gene silencing in MDA-MB-468 cells.** (A) Relative AKT2 mRNA expression and (B) Western blot analysis of AKT2 protein levels in MDA-MB-468 cells treated with heterotypic PLL/TNA_AKT2_@231CM NPs. No significant knockdown is observed, confirming the requirement of homotypic membrane coating for effective gene silencing in drug-resistant cells.


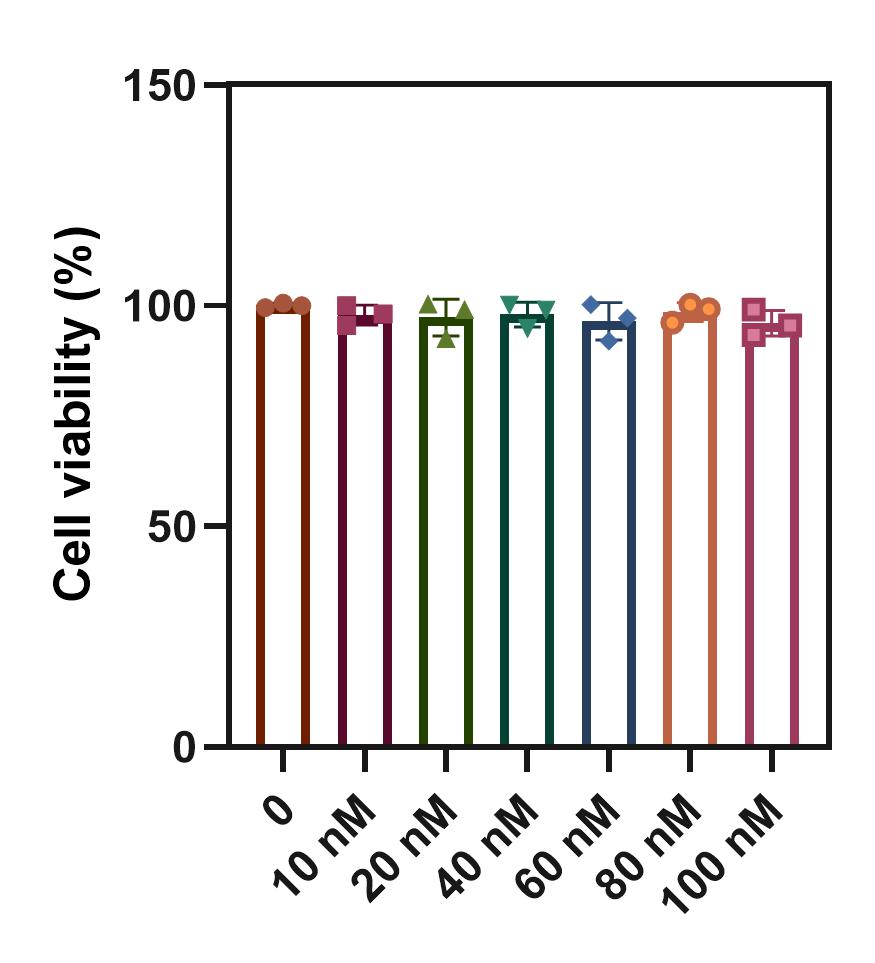


**Figure S9. Biocompatibility evaluation of PLL polymer in MDA-MB-468 cells.** Cell viability measured by MTT assay after 48 h incubation with increasing concentrations of PLL. High cell viability (>95%) is maintained even at 100 nM, demonstrating excellent biocompatibility of the PLL carrier.


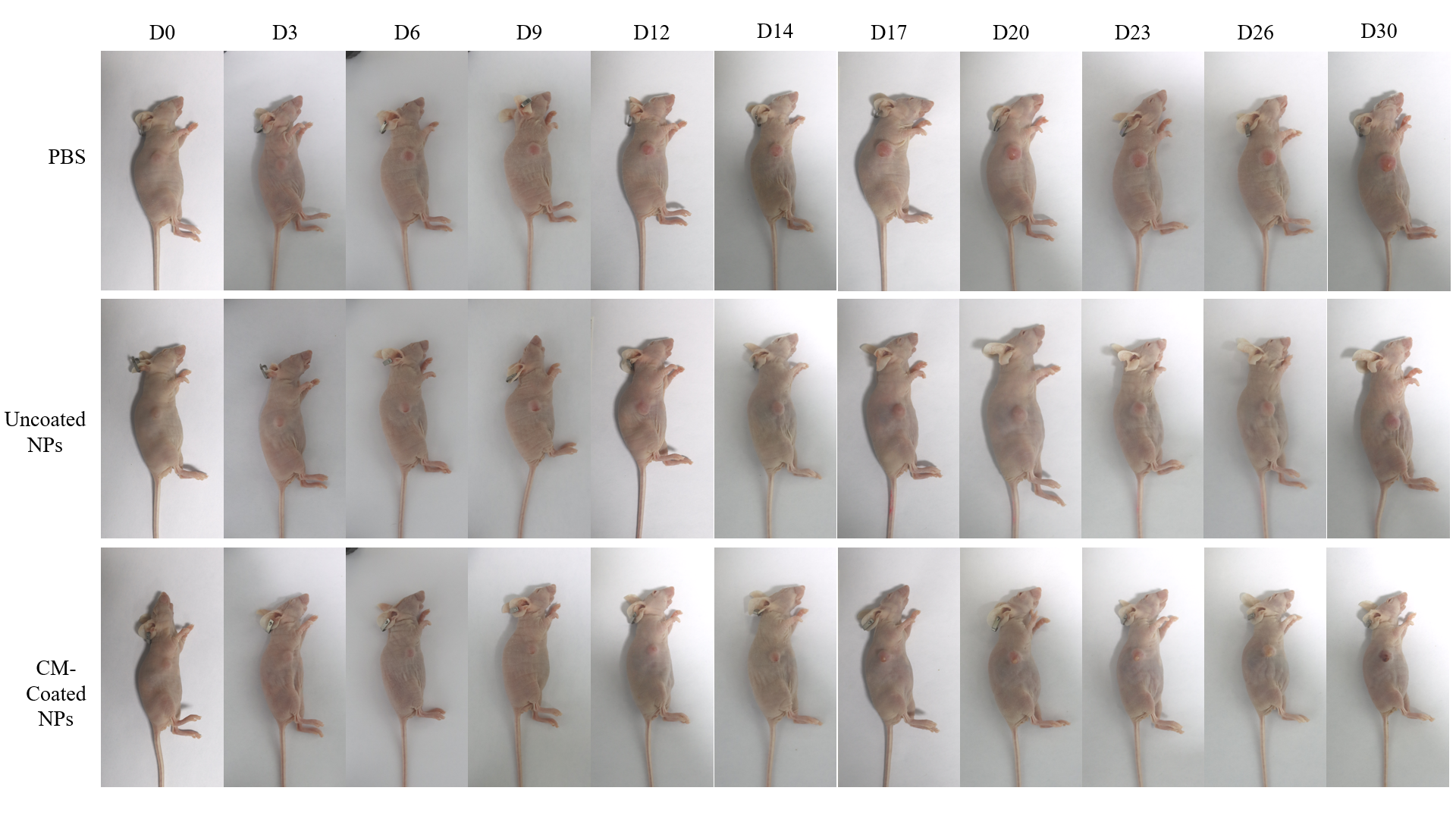


**Figure S10.** Photographs of MDA-MB-468 tumor-bearing nude mice every three days.


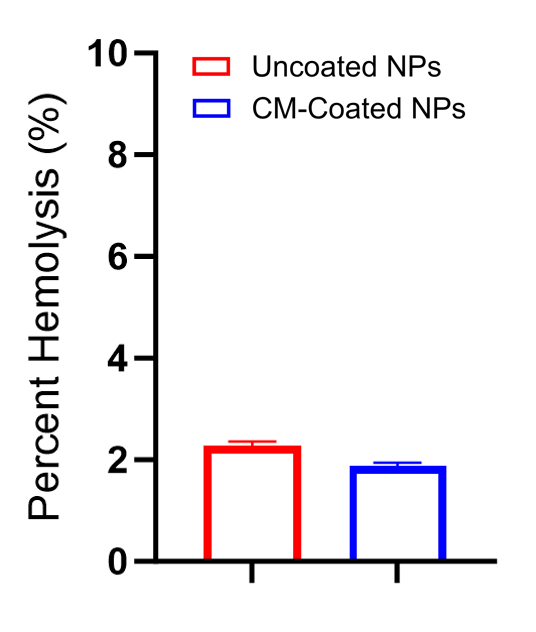


**Figure S11**. Percent hemolysis of uncoated NPs and CM-Coated NPs.

**Video S1-2.** Real-time membrane fusion of PLL/TNA_AKT2_@468CM NPs in MDA-MB-468 cells. These time-lapse videos (captured 55–60 min post-incubation) demonstrates the internalization of DiO-labeled PLL/TNA_AKT2_@468CM NPs (donor) by DiI-labeled MDA-MB-468 cells (acceptor). The emergence of a robust, sensitized DiI emission (visualized as the FRET signal in Video 1 and the merged signal in Video 2) at the cell periphery provides real-time spatiotemporal evidence of direct lipid mixing. These results confirms that the biomimetic nanoparticles bypass traditional endocytic pathways via a rapid membrane-fusion mechanism, thereby facilitating direct cytosolic delivery of the TNA cargo.

**Video S3-4:** Inhibition of membrane fusion via MβCD-mediated cholesterol depletion. These time-lapse videos (captured 55–60 min post-incubation) shows MDA-MB-468 cells pre-treated with the cholesterol-depleting agent MβCD prior to incubation with DiO-labeled PLL/TNA_AKT2_@468CM NPs. In stark contrast to the untreated cells in Video S1,a negligible FRET signal is observed (Video 3). Instead, discrete DiI and DiO signals remain spatially separated at the cell periphery and within the PLL/TNA_AKT2_@468CM NPs, respectively (Video S4). These results confirm that membrane cholesterol is a critical mediator of the fusion process; its depletion significantly hinders the ability of the biomimetic shell to fuse with the host plasma membrane, preventing direct cytosolic entry.
